# The Therapeutic Efficacy of Dendrimer and Micelle Formulations for Breast Cancer Treatment

**DOI:** 10.3390/pharmaceutics12121212

**Published:** 2020-12-15

**Authors:** Sibusiso Alven, Blessing Atim Aderibigbe

**Affiliations:** Department of Chemistry, University of Fort Hare, Alice Campus, Eastern Cape 5700, South Africa; 201214199@ufh.ac.za

**Keywords:** breast cancer, micelles, dendrimers, anticancer drugs, drug delivery, doxorubicin, platinum drug, methotrexate

## Abstract

Breast cancer is among the most common types of cancer in women and it is the cause of a high rate of mortality globally. The use of anticancer drugs is the standard treatment approach used for this type of cancer. However, most of these drugs are limited by multi-drug resistance, drug toxicity, poor drug bioavailability, low water solubility, poor pharmacokinetics, etc. To overcome multi-drug resistance, combinations of two or more anticancer drugs are used. However, the combination of two or more anticancer drugs produce toxic side effects. Micelles and dendrimers are promising drug delivery systems that can overcome the limitations associated with the currently used anticancer drugs. They have the capability to overcome drug resistance, reduce drug toxicity, improve the drug solubility and bioavailability. Different classes of anticancer drugs have been loaded into micelles and dendrimers, resulting in targeted drug delivery, sustained drug release mechanism, increased cellular uptake, reduced toxic side effects of the loaded drugs with enhanced anticancer activity in vitro and in vivo. This review article reports the biological outcomes of dendrimers and micelles loaded with different known anticancer agents on breast cancer in vitro and in vivo.

## 1. Introduction

Cancer is a life-threatening disease characterized by abnormal and uncontrolled cell proliferation, which can invade other organs of the body [1]. Approximately 90–95% of cancer cases are attributed to genetic mutation and 5–10% cases are caused by inherited genetic mutations [2]. The World Health Organisation (WHO) reported 9 million cancer-related deaths in 2018, which occurred mostly in Asia and Africa. Types of cancer are classified based on the body organ affected, and some examples of cancer types include breast, lung, liver, colorectal, skin, brain, stomach, and pancreatic cancer, etc. [3,4].

Among the cancer types, breast cancer is common in women, and it has been reported to be the cause of approximately 627,000 deaths in the world annually [5]. There were 230,000 breast cancer cases diagnosed in the United States alone with over 40,000 deaths, and it is identified as the second common cause of cancer-related death in women [6]. The mortality rate relating to breast cancer has decreased over the past 20 years by 30% due to consistent progress in drug development, which has resulted in an improved survival rate of 90% [7]. Despite the improvements in the survival rate in some developed countries, metastatic breast tumor is still challenging to treat with a predictable overall survival rate of only 23% [8]. The main pathways that are involved in breast cancer metastases and development are not well understood.

Breast cancer is classified into three subtypes based on the occurrence and absence of three receptors present in the tumor cells: (i). hormone receptor (HR) positive breast cancer (express progesterone and estrogen receptors), (ii). Oncogene human epidermal growth factor 2 (HER-2/neu), and (iii). Triple-negative breast cancer (TNBC) (negative for expression of progesterone, estrogen, and HER-2/neu) [9,10]. The TNBC is the worst chronic breast tumor subtype. Several methods used to treat cancer include chemotherapy, hormonal therapy, radiotherapy, immunotherapy, and surgery [11]. Chemotherapy is one of the best treatment methods used to combat breast cancer subtypes, although it suffers from several shortcomings [12].

Chemotherapy is the most utilized approach, and recently it involves the combination of two or more anticancer agents to overcome the development of drug resistance. The shortcomings of chemotherapy include multi-drug resistance, drug toxicity, poor drug bioavailability, low water solubility, and poor pharmacokinetic parameters [13]. Polymeric nanocarriers are effective potential systems that can be utilized for targeted drug delivery. Their capability to overcome the shortcomings of anticancer drugs that are associated with chemotherapy is promising. They are drug delivery systems formulated from polymer-based materials and are used to deliver different types of bioactive agents (e.g., hydrophilic and hydrophobic drugs) to the targeted biological environment. There are various types of polymeric nanocarriers utilized for the delivery of bioactive agents resulting in improved therapeutic outcomes such as polymeric nanoparticles [14,15,16,17,18], nanoliposomes [19,20,21,22], nanocapsules [23], polymer prodrugs [24,25,26,27,28,29], nanogels [30], hydrogels [31], dendrimers [32,33], and micelles [34,35,36,37]. Polymeric nanocarriers offer several unique and potent advantages: improved drug solubility, enhanced drug biodegradability, bioavailability, and biocompatibility, improved patient compliance, controlled and sustained drug release kinetics, preservation of the drug efficacy during plasma circulation, reduced drug toxicity, and overcome drug resistance [34,38]. Due to the unique features of polymeric nanocarriers, this review will report the in vitro and in vivo biological efficacy of micelles and dendrimers designed for the treatment of breast cancer.

## 2. Breast Cancer and Its Chemotherapy

Among the subtypes of breast cancer, 60% of breast cancer cases are HR-positive breast tumors, and HER-2/neu constitute around 20% of all breast cancer cases. Approximately 20% of breast cancer cases are TNBC [39]. Breast cancer-related symptoms include a change in the shape and size of breast, skin dimpling, swollen lymph nodes, red patch of the skin, a lump in the breast, discharge of fluid from the nipple, bone pain, and shortness of breath [40]. Several factors that contribute to breast cancer include obesity, early age menstruation, smoking, inherited genes mutations, ionizing radiation, lack of exercise, etc. [41]. Breast cancer therapies presently used are chemotherapy, radiation therapy, immunotherapy, surgery, hormone therapy, and antibody therapy [42,43]. These therapies have short and long-term consequences that affect patients’ quality of life [43].

**Figure 1:**
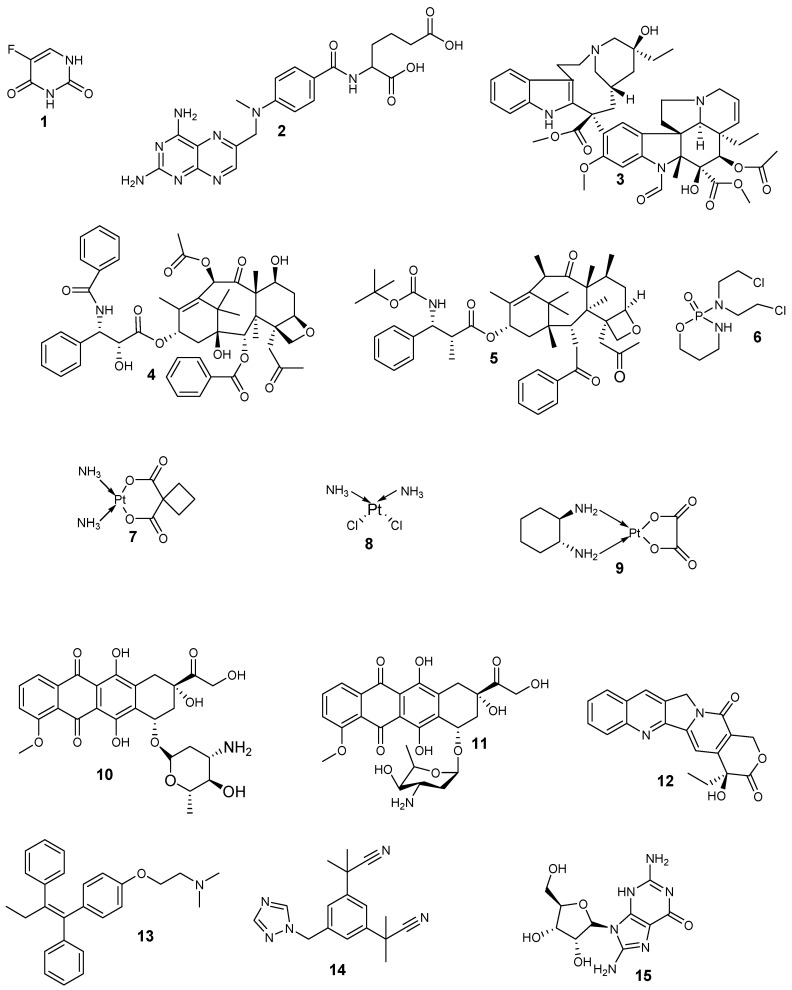
Anticancer drugs used for the treatment of breast cancer: fluorouracil (**1**), methotrexate (**2**), vincristine (**3**), paclitaxel (**4**), docetaxel (**5**), cytoxan (**6**), carboplatin (**7**), cisplatin (**8**), oxaliplatin (**9**), epirubicin (**10**), doxorubicin (**11**), camptothecin (**12)**, tamoxifen (**13**), arimidex (**14**) and trastuzumab (**15**).

Anticancer drugs (Figure 1, Table 1) act by interfering with the abnormal, uncontrolled cell division and are cell cycle phase non-specific or specific [43,44]. Cell cycle phase-specific bioactive agents include the plant alkaloids (e.g., vinca alkaloids and taxanes) and the antimetabolites (e.g., fluorouracil (**1**), methotrexate (**2**)) [45]. Antimetabolites are very effective in the S-phase of cell division, and they act specifically on cells involved in the formation of new cells from the synthesis of new DNA. Plant alkaloids are effective in the S- and M-phases. Fluorouracil acts as a thymidylate synthase inhibitor by interfering with DNA replication. Vinca alkaloids (e.g., vincristine (**3**)) bind with microtubule assembly, destroying chromosome segregation during mitosis. The taxanes (e.g., paclitaxel (**4**), docetaxel (**5**)) prevent the normal functioning of microtubule during mitosis and hinders cell division [45].

Alkylating agents are cell cycle phase non-specific bioactive agents, and their examples include cyclophosphamide (cytoxan (**6**)), they target the replicating cells’ DNA. Other examples of alkylating agents are platinum drugs (e.g., carboplatin (**7**), cisplatin (**8**) and oxaliplatin (**9**)) which are responsible for the inhibition of DNA synthesis in cancer cells. Anti-tumor antibiotics are also cell cycle phase non-specific bioactive agents (e.g., epirubicin (**10**), doxorubicin (**11**) and camptothecin (**12**)). They prevent RNA (ribonucleic acid) synthesis by binding with the DNA, thereby distorting the DNA structure [46]. Other chemotherapeutic agents employed for breast tumor chemotherapy include tamoxifen (**13**) a selective estrogen-receptor modulator) administered to the patient with ER-positive breast tumor, and it acts as an estrogen antagonist. Another drug used to treat breast cancer is arimidex (**14**) which hinders aromatase action. It is administered to postmenopausal women diagnosed with hormone-receptor-positive, metastatic hormone-receptor-positive breast cancer, and advanced-stage breast cancer. Trastuzumab ((**15**), a biological response modifier) acts as a HER2 protein inhibitor on breast cancer cells, terminate cell division, and causes apoptosis and cell stasis [46].

All the aforementioned anticancer drugs can affect healthy cells (e.g., bone marrow, and hair follicle, etc.), resulting in significant and frequent adverse effects that include alopecia, loss of appetite, vomiting, and nausea, thrombocytopenia, neuropathy, mucositis, myelosuppression-induced anemia, etc. [47]. The chemotherapeutic drugs are employed to target the rapid division of cancer cells; various cells in the human body rapidly divide in a normal manner, such as the digestive tract cells and hair follicle cells, which are also affected by the chemotherapeutics. Also, the widespread distribution and short half-life of anticancer drugs require more dosing, resulting in increased side effects [47]. The anticancer drugs’ side effects are due to their non-targeted and non-specific nature that result in the healthy cells/tissue exposure to their toxic side effects. Polymeric nanocarriers are promising candidates that can be employed to overcome the shortcomings of currently used anticancer drugs. Hence this review article is focused on polymeric micelles and dendrimers nanocarriers loaded with anticancer drugs for breast tumor targeting.

## 3. Micelles and Dendrimers Nanoformulations That Are Currently in the Clinical Trials

Micelles display excellent features such as good tumor-targeted delivery, making them suitable as a drug delivery system with high translational potential. Some micelle-based systems are presently under various phases of clinical assessment (Table 2), but most of them are still under preclinical evaluation [48]. Genexol^®^-PM and NK105 were developed to improve the water solubility and therapeutic efficacy of paclitaxel and irinotecan (topoisomerase-I inhibitor). Genexol^®^-PM is known as the copolymer micelle made up of mPEG-PDLLA solubilizer. Its enhanced water solubilizing efficiency of 25% is higher than the formulation, Taxol^®^. In addition, this micelle formulation has a greater maximum tolerated dose when compared to Taxol^®^ [48]. Furthermore, Genexol^®^-PM exhibits higher cellular internalization and good inhibition of P-glycoprotein, and decreased myelo-suppression when compared to Taxol^®^. Presently, Genexol^®^-PM has been approved for clinical application in South Korea, Hungary, and Bulgaria, and it is being assessed in Phase II clinical trials in the USA. It is sold under the trade name Cynviloq™ [48,49].

NK105 is a micelle formulation developed from PEG-poly(aspartic acid) copolymer and 4-phenyl-1-butanol to improve its hydrophilicity. It delivers loaded paclitaxel to solid tumors [50]. Tumor maximum concentration of these micelles was higher than paclitaxel when screened on HT-29 colon tumor model. Nippon Kayaku Co. Ltd. (Tokyo, Japan), a certified company in Japan, began phase II clinical trial of NK105 [50]. NK012 micelle formulation is composed of conjugated drug SN38 (irinotecan hydrochloride) with poly(l-glutamic acid) fragment of PEG–P(Glu) block copolymer. Despite its significant cytotoxicity against several cancer cell lines screened, NK012 micelle is an effective micellar system with an average particle size of 20 nm and the drug loading capacity of approximately 20% [51]. Two clinical trial phases were completed independently in the USA and Japan, with no serious dose-dependent side effects. Based on these findings, NK012 micelles are currently under phase II clinical trial [51].

The micelle products NC-6300, NK911, and NC-6004 are formulated for the delivery of epirubicin, doxorubicin, and cisplatin, respectively [52]. The antitumor activity of NC-6300 with a significant decrease in cardiac toxicity in the phase I clinical evaluation in 2013 revealed that the formulation is safe and tolerable [53]. NK911 formulation in a clinical trial at the National Cancer Centre Hospital in Japan, Tokyo, showed the recommended dose and minimum toxicity dose of 50 and 67 mg/m^2^, respectively [53]. The NC-4016 micelles formulation containing DACH-platinum was reported to be stable in physiological environments with prolonged blood plasma circulation after bolus administration resulting in more than 1000-fold increase in blood plasma concentration between 0 to 3 days when compared to free oxaliplatin during preclinical evaluations. Phase I clinical study commenced in 2013 at the University of Texas MD Anderson Cancer Centre in the USA in patients with advanced lymphoma or solid tumor [54]. NC-6004 (Nanoplatin™) was evaluated at phase I/II clinical trial in patients with advanced solid tumour to evaluate its safe dose, safety, efficacy, and tolerability [55]. Seventeen patients with advanced solid tumour types were administered the formulation intravenously with a starting dose of 10 mg m^−2^ which was increased to 120 mg/m^2^. The maximum tolerated dose of 120 mg/m^2^ and a recommended dose of 90 mg/m^2^ was reported. The sustained release of cisplatin after administration contributed to the low toxicity of the formulation.

SP1049C micellar formulation has been reported to overcome multidrug resistance in a clinical trial [56]. It is composed of Pluronic L61 and Pluronic F127. It is loaded with doxorubicin. Pluronic L61 inhibits the efflux mechanism of P-glycoprotein, while pluronic F127 enhances the stability of the formulation. The biodistribution of the formulation was similar to the free doxorubicin in the liver, blood plasma, kidney, lungs, and heart. The cytotoxicity of the formulation was significant against a brain tumor. Its effective therapeutic outcomes were reported in preclinical studies, phase I and II clinical trials in patients with advanced adenocarcinoma of gastroesophageal and oesophagus junctions. It is a registered chemotherapeutic agent for the treatment of metastatic adenocarcinoma [56].

On the other hand, the use of dendrimer-based nanocarriers for cancer therapy is beneficial. A lot is left to be discovered and addressed to confirm their efficacy in cancer treatment [57]. One of the antitumor dendrimer formulations that have succeeded from preclinical evaluation to Phase I clinical study is DEP^®^ docetaxel. DEP^®^ docetaxel formulated from PEGylated PLL dendrimer has shown promising results in clinical trials revealing the efficacy of dendrimer systems [58]. Other dendrimers currently under clinical studies are DEP^®^cabazitaxel, ImDendrim, and MAG-Tn3 [59].

## 4. Dendrimers for Breast Cancer Therapy

**Figure 2:**
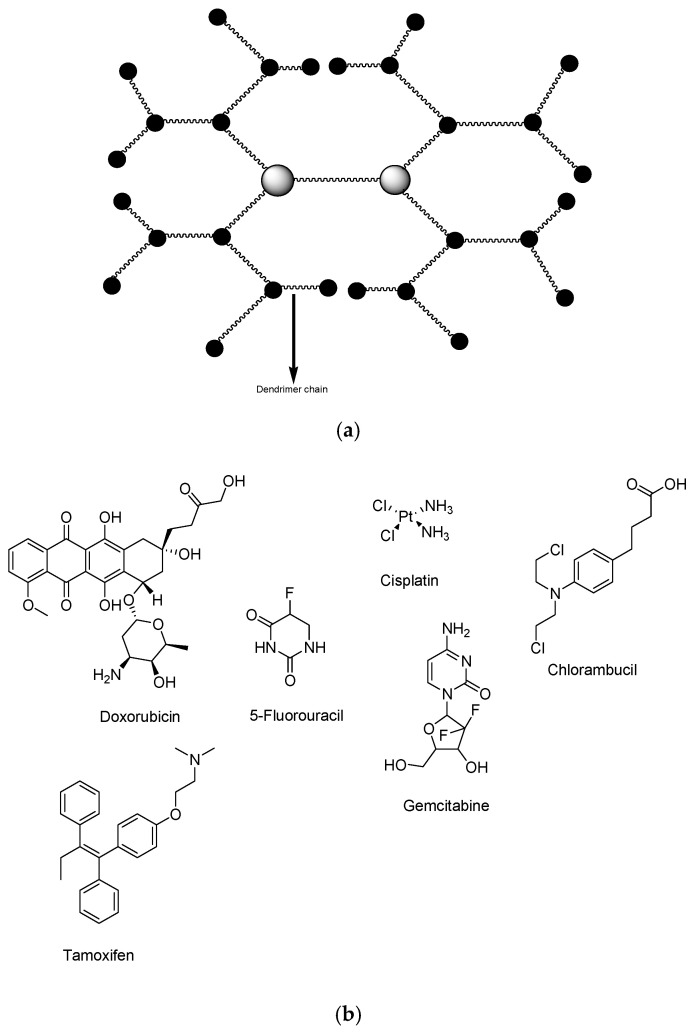

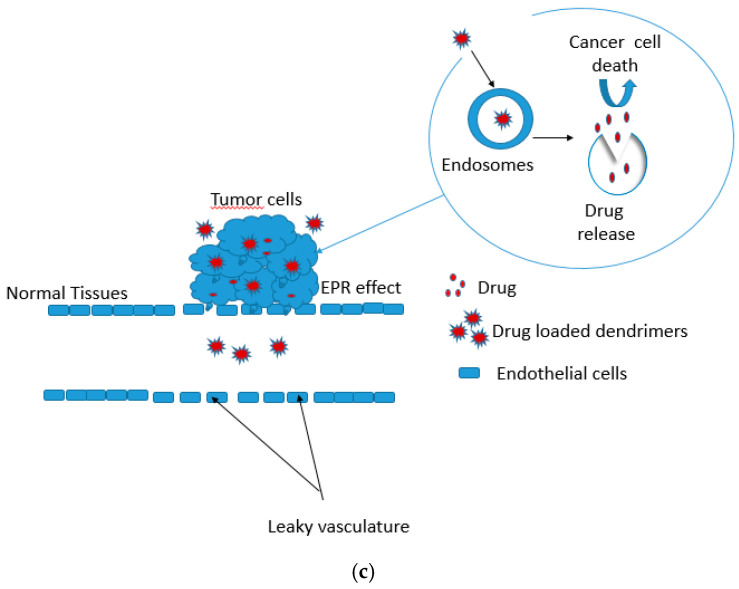
(**a**): A schematic diagram of a dendrimer. (**b**): Structures of some anticancer drugs which have been loaded into dendrimers. (**c**): Drug uptake from dendrimer into tumor cells via EPR effect.

Dendrimers are nanocarriers with hyperbranched, spherical, and three-dimensional (Figure 2a). Different anticancer drugs have been loaded into dendrimers for the treatment of breast cancer (Figure 2b) (Table 3) [60,61]. They are utilized in biomedical applications for drug delivery of bioactive agents such as antimalarial, anticancer, antiviral, antiprotozoal, antitubercular drugs, etc. Their well-defined surface functional groups and globular nanosize in the range of 1–10 nm makes them useful for drug delivery [62]. Polymeric dendrimer is of great interest in biomedical applications such as polyamidoamine (PAMAM) dendrimers due to their low toxicity [62,63]. These nanocarriers have successfully delivered chemotherapeutic agents and in theranostic applications in chemotherapy [64]. Other types of dendrimers used in biomedical applications, especially in oncology include poly-lysine, PPI, phosphorus, and carbosilane dendrimers. Polylysine (PLL) is one of the amphiphilic dendrimers with branched structure based on penta-functional core molecules obtained by functionalization of positively charged basic amino acids like lysine or β-amino-alanine [65]. PLL demonstrates a remarkable new group of molecules since they have a small size and are made of natural components that are more easily internalized than those based on synthetic molecules. Cationic PLL dendrimers are useful in numerous biomedical applications, such as carriers for antitumor drugs (5-fluorouracil etc.) [65]. Poly (propyleneimine) (PPI) dendrimers are highly branched macromolecules with symmetric architecture, and they have been widely studied for DNA delivery. These dendrimers can enhance transfection efficiency by endocytosis and direct transport into the cell nucleus [66]. Phosphorous and carbosilane dendrimers are inorganic dendrimers with interesting biomedical applications. These dendrimers demonstrate therapeutic effects against many cancer types and can be loaded with different anticancer drugs for drug delivery [67,68].

The other advantages of dendrimers in chemotherapy include enhanced drug efficacy, improved drug biocompatibility, reduced drug toxicity, and controlled and sustained drug release kinetics [69,70,71,72].

### 4.1. Dendrimers Loaded with Doxorubicin 

Dendrimers can overcome the toxic side effects of doxorubicin by promoting sustained release profile (Table 3). Guo and co-workers formulated hyaluronic acid (HA)-modified amine-terminated fourth-generation PAMAM dendrimers for co-delivery of doxorubicin and cisplatin [73]. The TEM images of the drug-loaded dendrimers displayed spherical shaped morphology. The dual drug-loaded dendrimers displayed a mean particle size of 91 ± 2.18 nm, a negative surface charge of −24 ± 7.6 mV, and a polydispersity index (PDI) of 0.35 ± 0.06. The dendrimers’ drug loading efficiency was 13.6% and 18.3% for cisplatin and doxorubicin, respectively [73]. The drug release studies of PAMAM dendrimers in an acidic tumor condition and physiological environment (pH 7.4) in vitro showed no release of both drugs within 2 and 24 h, respectively, demonstrating that co-loaded dendrimers were sufficiently stable to avoid drug clearance from the blood plasma before reaching the target tissue. The cellular uptake studies of the formulation in MDA-MB-231 and MCF-7 breast cancer cell models using confocal scanning microscopy showed that the cellular uptake was time-dependent. The antitumor analysis in vitro of HA modified co-loaded PAMAM dendrimers and plain co-loaded PAMAM dendrimers were evaluated on both cancer cell lines using MTT assay. The modified co-loaded drug dendrimers showed good anticancer efficacy of 58% cell death while the plain co-loaded dendrimers showed 49% cell death on MDA-MB-231 cells [73]. PAMAM dendrimers uptake into cancer cells is via a clathrin-mediated uptake passageway followed by rapid transport to the cellular compartments such as (endosomes and lysosomes) [74]. The modified dual drug-loaded dendrimers were stable, and their uptake was via the lysosome-mediated pathway. It also enhanced drug accumulation in the tumor tissue when compared to the free drug solutions. After 24 h of distribution studies, no drugs were observed in the kidneys and the heart, revealing the reduced side effects of the formulation. The in vivo antitumor studies in tumor-bearing BALB/c mice showed that the modified dual drug-loaded dendrimer formulation exhibited significant antitumor effect. The study revealed the synergistic efficacy of co-delivery of anticancer drugs using dendrimers [73].

Incorporating doxorubicin into dendrimers can also result in targeted drug delivery to the cancer cells, and the nature of the targeting moiety plays an important role. Chittasupho et al. prepared CXCR4 targeted dendrimers encapsulated with an antitumor antibiotic, doxorubicin, using PAMAM dendrimers [75]. The encapsulation and drug loading capacity of doxorubicin in the PAMAM dendrimers were 97.25 ± 0.04 and 3.40 ± 0.04%, respectively. The in vitro drug release kinetics of the doxorubicin from the dendrimers was quick at pH 5.0 compared to pH 7.4. After 2 h of treatment, the uptake of the formulation into BT-549-Luc cells was 10.7 (for 0.25 mg/mL), 11.5 (for 0.5 mg/mL), and 13.2 (for 1 mg/mL) fold higher when compared to the dendrimer without a targeting moiety. The uptake of the formulation into T47D cells were 3.0 (0.25 mg/mL), 4.6 (0.5 mg/mL), and 4.3 (1 mg/mL)-fold higher when compared to the dendrimer without a targeting moiety. The IC_50_ values of the drug-loaded dendrimer on BT-549 after 12 h were 25.2 μg/mL compared to the free drug, which was 72.6 μg/mL. The free drug did not display any cytotoxic effect on the T47D cells, but the IC_50_ values of the drug-loaded dendrimer on the cell lines after 120 h was 124.4 μg/mL [75]. The cellular uptake of the dendrimers into T47D and BT-549-Luc breast tumor cell was time and concentration-dependent. The cytotoxicity of the doxorubicin-loaded polymeric dendrimer was potent compared to the free doxorubicin and unloaded dendrimers. The cyclic pentapeptide, FC131 [(cyclo)(d-Tyr-Arg-Arg-l-3-(2-naphthyl)alanine-Gly)] incorporated into the formulation is a potent antagonist of CXCR4, an important chemokine receptor that is involved in the metastasis of cancers. The presence of FC131 in the formulation inhibited BT-549-Luc cells migration resulting from reduced interactions between SDF-1α and CXCR4 receptors [75].

Dendrimers are also potent systems to combat multi-drug resistant breast cancer. Wang et al. formulated pluronic F68-incorporated PAMAM dendrimer conjugates loaded with doxorubicin. The in vivo and in vitro anticancer studies revealed doxorubicin pluronic F68-PAMAM dendrimers increased antitumor efficacy against MCF-7/ADR cancer cells by caveolae-mediated endocytosis. They significantly increased apoptosis by regulating gene expression and mitochondrial function [76]. The use of pH-sensitive linkers for the incorporation of drugs to dendrimers is a promising approach. Kojima et al. prepared collagen peptide-modified dendrimers encapsulated with doxorubicin for breast cancer treatment. The drug was incorporated via a pH degradable linker. The diffusion of the drug from the dendrimers was reduced. The reduced release of the drug is effective in overcoming drug resistance. The dendrimers exhibited significant anticancer efficacy against MCF-7 and MDA-MB-231 breast cancer cells with in vivo attenuated metastatic activity. MDA-MB-231 cells were more sensitive to the formulation when compared to MCF-7 cells. The dendrimer inhibited tumor growth significantly [77].

Similar findings on the effect of the linkers on the release profile of DOX was also reported by Kaminskas et al. [78]. Generation 5 PEGylated polylysine dendrimers composed of an outer generation of l-lysine or succinimyldipropyldiamine (SPN) loaded with doxorubicin via 4-(hydrazinosulfonyl) benzoic acid (HSBA) linker. The release of DOX from the formulation was slow with a release of less than 10% of DOX in pH 7.4 buffer for 3 days. However, a 100% release of DOX was significant in pH 5. The formulation retained the cytotoxic properties of the loaded DOX in vitro. The clearance patterns of both the DOX conjugated dendrimers were similar to the free DOX. However, the SPN dendrimers showed reduced metabolic lability and increased uptake into RES organs compared to the equivalent all-lysine dendrimers. In vivo studies of the formulations in rats bearing Walker 256 tumours revealed a high uptake into the tumour tissue. The labile HSBA linker influenced the targeted drug delivery mechanism of DOX to tumours [78]. The size of the dendrimers is also crucial in their therapeutic outcomes. Mehta et al. investigated the influence of dendrimer generation (G4 and G5) and PEG lengths (of 570 and 1100 Da) on selected factors such as the pharmacokinetics, drug release kinetics, tumor biodistribution, and anticancer activity. The PEGylated polylysine dendrimers were conjugated with doxorubicin using a cathepsin-B cleavable valine-citrulline linker. The largest G5 PEG1100 dendrimer good tumor and retention was good but the drug release was slow, thereby limiting its anticancer activity. The smallest G4 PEG570 dendrimer was significantly efficient in cathepsin-mediated doxorubicin release, but its systemic exposure and tumor uptake were limited. The intermediate-sized dendrimer displayed better drug release kinetics, tumor uptake, systemic exposure, and good retention. These findings revealed the influence of the PEG molecular weight and dendrimer size on the therapeutic efficacy of the dendrimer formulations [79].

Dox is passively transported via the cellular membrane, which makes it prone to efflux pumps [80]. It is not specifically targeted to the tumour but affects the growth of other cell types in the body, thereby resulting in a compromised immune system. The use of dendrimers for the delivery of DOX offers several advantages, such as prevent drug clearance from the blood plasma before reaching the target tissue [73,75]. It also enhances drug accumulation in the tumor tissues and inhibits drug distribution to healthy tissues/organs [73,78]. The linkers used to incorporate DOX influenced its rate of release from the formulation and its uptake into cancer tissues [75,77,78,79]. The dendrimer formulations loaded with DOX were specific and effective against some breast cancer cell lines [77]. Generally, the release of DOX from the dendrimer formulations was enhanced at acidic pH [73]. The dendrimer size also plays a crucial role in the therapeutic efficacy of dendrimer formulations [79].

### 4.2. Dendrimers Loaded with Oligodeoxynucleotides

Gene delivery using dendrimers have been reported to be an effective approach resulting in enhanced biological outcomes (Table 3). Wang and co-workers synthesized G4 PAMAM dendrimers loaded with antisense oligodeoxynucleotides for gene delivery in the treatment of breast cancer. Incorporating antisense oligodeoxynucleotides to the dendrimers reduced its toxicity, enhanced its stability binding and inhibits its capability to bind to the erythrocytes and BSA. The cellular uptake of the antisense oligodeoxynucleotides from the dendrimers was high in MDA-MB-231 breast tumor cells. In vivo anticancer experiment using human breast tumor xenograft mice model showed that these dendrimers possess a high capacity of accumulating antisense oligodeoxynucleotides, thereby hindering the tumor vascularization [81]. Similar high cellular uptake of dendrimer-based formulations was also reported by Chen and co-workers. Breast cancer cellular uptake of oligodeoxynucleotide nanoparticles formed in the presence of polypropyleneimine dendrimers was studied. The confocal microscopy results demonstrated higher cellular uptake of oligodeoxynucleotide loaded dendrimers by MDA-MB-231 breast cancer cells compared to free oligodeoxynucleotide. The cellular uptake of G-4 and G-5 dendrimers by the breast cancer cell (MDA-MB-231) was facile. G-1 to G-3 dendrimers exhibited lower zeta potential (5.2–6.5 mV) compared to G-4 and G-5 dendrimers which were in the range of (12–18 mV). The cellular uptake of dendrimers was facile, suggesting that the structure and charge density are essential in cellular transport [82].

Furthermore, the coating of dendrimers promotes targeted drug delivery, thereby inhibiting rapid drug clearance. Pourianazar and Gunduz formulated PAMAM dendrimer-coated magnetic nanoparticles entrapped with CpG oligodeoxynucleotide for breast cancer treatment. The particle size analysis displayed an average particle size of approximately 40 ± 10 nm. The cytotoxicity experiment of the dendrimer nanoparticles using MTT assay demonstrated induced cell death in SKBR3 and MDA-MB231 breast tumor cells, indicating that the dendrimers are suitable as targeted nanocarriers for the delivery of CpG oligodeoxynucleotide. The magnetic core of the dendrimer is ideal for targeted delivery. The formulation displayed high apoptotic capability. The loading of CpG- oligodeoxynucleotide into the magnetic nanoparticles is crucial for targeted delivery to the tumor, where the nanoparticles undergo endocytosis leading to their uptake in the endosomal sites. The mechanism mentioned earlier inhibits their rapid clearance by nucleases [83]. Xin and co-workers formulated G4 PAMAM dendrimers loaded with antisense oligodeoxynucleotide for breast cancer targeting. The G4 PAMAM dendrimers loaded with antisense oligodeoxynucleotide displayed a reduced copy number of Cyclooxygenase-2 mRNA and protein expression in the tumor tissue and the microvessel density in the tumor cells was also reduced with a significant tumor growth inhibition. The formulation displayed a high transfection rate, induced apoptosis with a G0/G1 cell cycle arrest [84].

Antisense and siRNA oligonucleotides are developed for the inhibition of target gene expression. Some dendrimers designed for Antisense and siRNA oligonucleotides incorporation display some toxicity in vitro on human erythrocytes [81]. However, the covalent linkage between G4PAMAM and antisense oligodeoxynucleotides enhanced the stability of dendrimer formulation with reduced toxicity. The dendrimers are useful in protecting therapeutic genes from destruction by enzymes, revealing the potency of dendrimers for transfection in gene manipulation [81]. The cellular uptake of dendrimers is attributed to their sizes and zeta potential. Dendrimers with significantly lower potential in the range of (5.2–6.5 mV) were not taken up by the breast cancer cells [82]. Loading CpG oligodeoxynucleotide in the magnetic core of the dendrimer resulted in targeted drug delivery with the capability to hinder rapid clearance [83]. G4 PAMAM dendrimers loaded with antisense oligodeoxynucleotide reduced the copy number of Cyclooxygenase-2 mRNA and protein expression in the tumor tissue and the microvessel density in the tumor cells resulting in a significant tumor growth inhibition [84].

### 4.3. Dendrimers Loaded with Trastuzumab

Dendrimers have also been designed for targeted delivery of trastuzumad (Table 3). Kulhari et al. formulated PAMAM dendrimers grafted with trastuzumab for targeted delivery to HER2-positive breast cancer. Dendrimers can cause hemolysis via binding to the erythrocytes via electrostatic interaction resulting in toxicity and reduced bioavailability. The in vitro drug release profile of the drug loaded dendrimers was sustained when compared to the control (Taxotere) over a period of 2 days. Hemolytic toxicity analysis revealed that trastuzumab-encapsulated dendrimers lowered hemolysis than the free dendrimers, showing concentration-dependent hemolysis. The cytotoxicity analysis in vitro of trastuzumab-encapsulated dendrimers showed low cell viability (approximately 36.2%) on MDA-MB-231 and MDA-MB-453 cancer cells when compared to those treated with docetaxel-loaded dendrimers (cell viability of approximately 57.6%) after 2 days [85]. Drug-loaded dendrimers display cell specificity, making them promising systems that can overcome drug toxicity. Miyano et al. prepared the G6 lysine dendrimers entrapped with trastuzumab for specific cellular internalization in HER2-positive breast cancer cells. The DLS analysis of the dendrimers displayed an average particle size of approximately 5–6 nm with negative zeta potentials and low PDI. The targeting efficiency showed that trastuzumab-loaded dendrimers bind specifically to SKBR3 breast cancer cells (HER2-positive) in a dose-dependent mode with a low binding affinity to MCF-7 cells (HER2-negative). The cellular internalization analysis demonstrated that these dendrimers were significantly internalized in SKBR3 breast tumor cells and then transferred to lysosomes [86]. Dendrimers are also designed for combination therapy with a combination of two anticancer drugs. Marcinkowska et al. developed PAMAM dendrimers for targeted binding to membrane receptors, HER-2 that are overexpressed in cancer cells. Over 20% of breast cancer displays an overexpression of HER-2 human epidermal growth factor receptor. The dendrimers were loaded with trastuzumab together with either docetaxel or paclitaxel via succinic acid linker. The formulation was highly toxic toward the HER-2-positive SKBR-3 cells but displayed low toxicity towards HER-2-negative MCF-7 cells. The accumulation of trastizumab was rapid in HER-2-positive SKBR-3 cell line when compared to the HER-2-negative MCF-7 cells which is influenced by the nature of incorporation via PEG linker into the dendrimer. However, a high amount of PAMAM- paclitaxel-trastuzumab dendrimer was significant in the HER-2-negative MCF-7 cells. The formulation selective binding of the PAMAM- docetaxel -trastuzumab conjugate on HER-2-positive SKBR-3 cells only was significant [87]. The pH-sensitive linker used made the formulation less stable in an acidic environment of the cancer cells. The dendrimer displayed high specific targeting to the HER-2-positive SKBR-3 cells. Trastuzumab, a recombinant, humanised IG1 monoclonal antibody, binds selectively to the human epidermal growth factor receptor 2 (EGFR2) and blocks the receptor hindering the uncontrolled proliferation of HER-2-positive cancer cells. The inhibition of the proliferation results in cell cycle arrest in the G1 phase making the combination of trastuzumab with other classes an attractive approach to develop potent anticancer drugs. The conjugation of dendrimer influences its biodistribution and specific targeting capability, thereby reducing toxicity. The combination of trastuzumad with neratinib using dendrimers is also effective against drug resistance and useful for targeted drug delivery. Aleanizy et al. formulated trastuzumab-grafted dendrimers loaded with neratinib for dual treatment of breast cancer to reduce drug resistance and promote targeted therapy [88]. Trastuzumab was conjugated to the surface of the dendrimer via maleimide-poly(ethylene) glycol-*N*-hydroxysuccinimide linker. In vitro analysis of the SKBR-3 cell viability after 48 h was 40%, 36%, and 33% for neratinib, neratinib-conjugated-dendrimers, and neratinib-loaded-dendrimers-trastuzumab, respectively. The affinity of trastuzumab to the HER2 receptors expressed in SKBR-3 cells promoted the internalization of the formulation via receptor-mediated endocytosis [88]. Chan and co-workers formulated diethylenetriaminepentaacetic acid-modified G4 PAMAM dendrimers conjugated with trastuzumab and nuclear translocation sequence (NTS) for breast cancer therapy. These dendrimers demonstrated retained HER2 immunoreactivity and were internalized in the nucleus of the breast cancer cells. The in vitro cytotoxicity analysis of G4 PAMAM dendrimers conjugated with trastuzumab and NTS exhibited potential anticancer activity against MDA-MB-231 and SK-Br-3 breast cancer cell lines [89]. Oddone and co-workers prepared and evaluated cellular uptake of PAMAM G4. 5 dendrimers loaded with florescein isothiocyanated in BALB/c mice breast tumors and murine breast cancer cells. The dynamic light scattering (DLS) analysis demonstrated the hydrodynamic particle size of 96.3 ± 1.4 nm with a PDI value of approximately 0.0296 ± 0.0171, and TEM results showed the particle size distribution of 44.2 ± 9.2 nm. The cellular uptake studies of PAMAM G4. 5 demonstrated significant uptake by the 4T1 cancer breast cells in vitro, and BALB/c mice breast tumors in vivo [90]. The type of drug conjugated to a dendrimer can influence its specific targeting capability. The conjugation of trastuzumab into dendrimers with selected classes of anticancer drugs can enhance its superior application for the treatment of some breast cancers due to the specific targeting mechanism into the tumor cells.

### 4.4. Dendrimers Loaded with Other Anticancer Drugs

Other anticancer drugs have been successfully loaded into dendrimers with good therapeutic outcomes on breast cancer (Table 3). Bielawski and co-workers formulated G3 PAMAM dendrimers loaded with chlorambucil [91]. Chlorambucil use is limited by its toxic side effects. The in vitro anticancer assessment revealed that the chlorambucil-entrapped dendrimers decreased the cell viability in both the estrogen receptor-negative (MDA-MB-231) and estrogen receptor-positive (MCF-7) breast tumor cell lines. The cytotoxic effect was concentration-dependent in both breast cancer cells. The dendrimers loaded with chlorambucil was more effective when compared to the free chlorambucil in both breast tumor models. The drug-loaded dendrimers IC_50_ values after 1 day of incubation in MDA-MB-231 and MCF-7 cells were 15 ± 2 nM and 25 ± 2 nM respectively, when compared to the free drug, which was 86 ± 2 nM and 88 ± 2 nM, respectively on both cancer cell lines. The formulation displayed stronger inhibition of collagen biosynthesis when compared to the free drug, chlorambucil. One characteristic feature of breast cancer cells is their interaction with extracellular matrix proteins is deregulated. Collagen is useful for the integrity of the connective tissue. A decreased amount of collagen in extracellular matrix can promote the movement and invasion of neoplastic cells, thereby contributing to the inhibition of cell growth and also the induction of apoptosis [92]. Studies using annexin V-FITC detected apoptosis by a fluorescent microscopy assay. It showed that the formulation hindered cancer cell proliferation by increasing the number of necrotic and apoptotic cells [91].

The number of terminal branches, hydrophilicity, and the dendrimers size positively influence their therapeutic outcomes. Abdel-Rahman and Al-Abd synthesized thermo-responsive dendrimers composed of tetrabromohydroquinone as the core and anticancer drug with branched oligoethylene glycol [93]. The thermoresponsive behavior studies at room temperature showed that all the polymeric dendrimers were aqueous-soluble. The lower critical solution temperature (LCST) of the dendrimers was in the range of 28–36 °C. The anticancer efficacy of the dendrimers was evaluated in vitro on the MCF-7 cancer cell model employing SRB-U assay and all the dendrimers exhibited significant cytotoxic effect with IC_50_ values ranging between 1.1 and 25.4 µg/mL. The resistant fractions of MCF-7 breast cancer cells to the formulation was in the range of 1.97–11.22%. The most potent dendrimers displayed an IC_50_ value of 1.1 µg/mL. The cellular uptake mechanism of the formulation and its cytotoxic effect was induced by an increase in the number of terminal branches and the interaction of the dendrimer lipid bilayer [93]. Factors to be considered when designing dendrimers include hydrophilicity in which increasing the hydrophilicity of the formulation reduce their penetration via the cell membrane; increasing the number of terminal branches, increases the cytotoxic effect of the formulation against cancer cells; increasing the dendrimer size will decrease the penetration of the formulation via the cell membrane.

Dendrimers loaded with metal-based nanoparticles have also been reported to be suitable for MR/CT molecular imaging of breast cancer cells. The currently used clinically contrast agents suffer from limitations such as renal toxicity at high concentrations, short imaging time, and non-specificity. Li and co-workers designed multifunctional dendrimer-based gold nanoparticles modified with PEG monomethyl ether and gadolinium chelate for breast cancer therapy [94]. Dendrimer-based metal nanoparticles were effective as a dual-modality contrast agent for MR/CT molecular imaging of breast cancer cells in vitro and in vivo. They were also found to be non-cytotoxic when used at a concentration of 0–50 μM. Their cellular uptake was efficient in vitro after incubation and in vivo in xenograft tumor model after intravenous injection of the formulation. The low uptake of gold nanoparticles in the liver and kidney indicates the capability of the NPs to escape the reticuloendothelial system in the liver and pass via the renal filter, thereby promoting an efficient uptake of the particles into the tumor by a passive EPR effect. The high uptake of the gold particle in the tumor region up to 0.276 g/kg ± 0.006 g/kg after 1 h of intravenous administration promoted effective MR/CT imaging of the tumors. Furthermore, the biodistribution of the formulation in the blood revealed their long blood circulation time with 0.0080 g/kg of the formulation in the blood 24 h after administration. The prolonged blood circulation time is attributed to the modification of PEG moieties on the dendrimer surface, which enhanced specific uptake by the reticuloendothelial system [94]. Despite the findings obtained, there is still a pressing need to evaluate the biodistribution behavior of the nanoparticles over an extended period of time.

Finlay et al. formulated PAMAM-RNA dendrimer complex to treat breast cancer by targeting TWIST1 transcription factor, which regularly is overexpressed in severe breast cancer. The cellular uptake of the siRNA loaded PAMAM dendrimers by the TNBC cells was effective, thereby causing significant inhibition of TWIST1 related target genes. Furthermore, the dendrimers’ capability to deliver siRNA to xenograft orthotopic tumor model was significant. siRNA was present in the tumor for over a period of four hours after treatment [95]. TWIST1 is a potentially clinically therapeutic target for the treatment of metastatic breast cancer [96]. However, the use of TWIST1 knockdown via PAMAM dendrimer-delivered siRNA is not suitable as a sole treatment for metastatic breast cancer. It can be used as an adjuvant therapy to reduce migration/invasion, chemoresistance, and antiapoptotic capability common with aggressive cancers.

Winnicka et al. formulated and evaluated the effect of G2 and G3 PAMAM dendrimers on human breast cancer cell lines. The in vitro anticancer study demonstrated that the G2 PMAMAM dendrimers possessed significant cytotoxic efficacy with an IC_50_ value of 140 ± 2 µM and 153 ± 3 µM against MDA-MB-231 and MCF-7 after 1 day of incubation and 99 ± 2µM and 120 ± 3 µM for G3 PAMAM, respectively. The inhibition in cell viability of the dendrimers was influenced by their apoptosis induction capability [97]. Dendrimers are useful in improving the water solubility of the incorporated drugs. Debnath et al. prepared dendrimers loaded with curcumin for the treatment of breast cancer. These dendrimers enhanced the water insolubility of curcumin. In vitro cytotoxicity analysis using MTT assay showed good anticancer activity of curcumin-loaded dendrimers against BT549 and SKBr3 breast cancer cell lines by inducing cellular apoptosis via caspase-3 activation [98]. The use of curcumin is limited by its poor water solubility and bioavailability. Incorporating it into dendrimers enhanced its water solubility with significant anticancer activity. Yao and co-workers designed polylysine dendrimer encapsulated with PHSCN peptide for breast cancer therapy. The in vitro anticancer analysis of peptide-loaded dendrimers exhibited a higher cytotoxic effect. In vivo, the formulation inhibited the extravasation of MDA-MB-231 and SUM-149 PT in the lungs of mice. PHSCN dendrimer was 700- to 1100-fold more effective than the PHSCN peptide, and they were effective in preventing the formation of metastatic colonies. The dendrimer capability to target the activated α5β1 integrins of the tumor cells without affecting the unactivated α5β1 receptors of healthy tissues indicate its potential to prolong the life span of patients with metastatic breast cancer [99]. The dendrimer displayed specific targeting capability. Lozano-Cruz et al. also reported loaded curcumin in the core of a “bow-tie” cationic carbosilane dendrimer. The dendrimers were highly soluble in water, retained the antioxidant activity of curcumin, and induced significant cytotoxic effect against MCF-7 cancer cells compared to the free curcumin [100]. The dendritic wedges played an important role in the anticancer and antioxidant activity of the dendrimers.

Winnicka et al. synthesized G3 PAMAM dendrimers incorporated with modified glycosides (proscillaridin A and digoxin) for the treatment of breast cancer. The in vitro cytotoxicity analysis of the dendrimers displayed improved anticancer efficacy against MDA-MB-231 and MCF-7 by the induction of significant cellular apoptosis compared to the apoptosis caused by the modified glycosides [101]. Mei et al. prepared PAMAM dendrimers co-loaded with 5-fluorouracil and antisense micro-RNA 21 gene for in vitro breast cancer cell suppression. micro-RNA 21 (miR-21) is overexpressed in breast cancer. The antisense inhibition of miRNA function is used to knockdown miRNA causing a significant inhibition of cell growth. The in vitro cytotoxicity results showed that the incorporation of micro-RNA 21 greatly enhanced the anticancer sensitivity of 5-fluorouracil in the MCF-7 cancer cells by effectively stimulating apoptosis and inhibiting the migration ability of MCF-7 breast cancer cells [102].

Zhang et al. synthesized PAMAM-NH_2_ dendrimers to reverse multidrug-resistant breast cancer cells (MCF-7/ADR cells). The in vitro cytotoxicity analysis of PAMAM-NH_2_ dendrimers demonstrated significant concentration-dependent toxicity with the cell viability being more than 85% at low concentration of (10–50 µg/mL) after 3 days, revealing the low anticancer effect of the dendrimer against MCF-7/ADR breast cancer cells at low concentration. The cell viability was decreased at a high PAMAM-NH_2_ concentration of 100–1000 µg/mL, confirming concentration-dependent toxicity [103]. P-gp and MDR-associated protein influenced higher PAMAM-NH_2_ exocytosis with lower PAMAM-NH_2_ endocytosis in the MCF-7/ADR cells than MCF-7 cells. The dendrimer degraded in the lysosomal vesicles of the MCF-7/ADR cells than in the MCF-7 cells. Dendrimers are promising systems that display high-efficiency transportation in sensitive and resistant cells.

Zhang and co-workers designed enzyme-responsive PEGylated lysine peptide dendrimers loaded with gemcitabine. The in vitro drug release profile of the dendrimers was significantly faster, with a release of approximately 80% gemcitabine in the tumor environment within 24 h. The drug release profile was influenced by the enzyme-cleavable linker, glycyl phenylalanyl leucyl glycine tetra-peptide used to conjugate the drug. The in vivo cytotoxicity experiment of gemcitabine loaded dendrimers using 4T1 murine breast cancer model indicated significant suppressed relative tumor volume of approximately 86.17 ± 38.27% and 2-fold higher tumor growth inhibition value of about 90% when compared to gemcitabine [104]. The nature of the linker used in the dendrimer influences their drug release profile.

Matai and Gopinath formulated hydrophobic myristic acid modified G5 PAMAM dendrimers for the delivery of tamoxifen in vitro to breast cancer cells [105]. The in vitro drug release profile at acidic condition (pH 5.5) of the tumor microenvironment showed sustained release of tamoxifen from the PAMAM dendrimer. Cellular uptake experiments showed that these dendrimers target the lysosome of the cancer cells. Furthermore, the anticancer analysis of dendrimers loaded with tamoxifen using MTT assay showed high inhibitory effects in MCF-7 human breast cancer cell lines [105]. The random grafting of lipid-like myristic acid chains to the surface of the dendrimers improved the stability and solubility of the loaded drug significantly. The myristoyl groups increased the cellular uptake and reduced the cytotoxicity of formulation.

Zhou et al. developed hyperbranched polyglycerol derivative (HPG-C18) and dendritic poly(l-lysine) for the codelivery of docetaxel and MMP-9 siRNA plasmid into tumor cells. The dendrimers were prepared by click reaction between azido-modified hyperbranched polyglycerol derivative and propargyl. The formulation displayed good gene delivery capability in vitro, which occurred via the induction of a decrease in MMP-9 protein expression in MCF-7 cells. The dendrimer displayed a significant apoptosis to breast cancer cells when compared to docetaxel or MMP-9. In vivo studies indicated that the codelivery of docetaxel or MMP-9 resulted in enhanced tumor inhibition [106].

### 4.5. Limitations of Dendrimers

The clinical translation of dendrimers is not rapid due to several limitations. Drugs are loaded into dendrimers via physical encapsulation or conjugation to the surface of the dendrimers [107]. Chemical conjugation can enhance drug loading, and the use of selective linkers results in targeted drug delivery. However, this drug loading approach can limit the availability of the drug that can be modified and can also significantly reduce the potency of the incorporated drug [108]. Furthermore, conjugating too many drugs on a dendrimer can increase the polydispersity index [73]. It also results in a slow drug release profile, which can reduce the efficacy of the conjugated drug. Guo et al. reported drug release studies of drug-loaded dendrimers in an acidic tumor condition and physiological environment in vitro and no release of both drugs within 2 and 24 h [73]. Although the finding demonstrates the high stability of the co-loaded dendrimers in blood plasma, the delayed drug release revealed that the linkers used did not promote rapid drug release in the tumor environment and could limit the anticancer activity of the formulation. Similar findings have been reported on the slow release of DOX and tamoxifen from the PAMAM dendrimer [77,78,105]. In another research report, a slow release of DOX from G5 PEG1100 dendrimer was reported to limit the anticancer activity of the formulation [79]. However, it is important to mention that the slow release of the drug from the dendrimers can overcome drug resistance and drug toxicity.

The physical encapsulation of drugs into dendrimer offers several advantages, such as ease of loading [109]. However, it often results in a large initial burst and inconsistent drug release, low stability upon storage, low-drug loading, premature drug release, etc. [108,109]. This is often reported as a rapid drug release in the first few hours [75]. PAMAMs are toxic, and their toxicity is overcome via modification of their structure. Dendrimers with cationic groups display significant toxicity when administered at high doses [108,110]. The surface charge of cationic dendrimers influences their strong interaction with anionic lipid bilayers by electrostatic interactions resulting in the formation of holes known as nanopores in the cell membranes with high cellular toxicity [111]. Furthermore, positively charged dendrimers have also been reported to be toxic which limits their use [112]. However, the surface modification of dendrimers has made them suitable for biological applications. Dendrimers such as poly(propylene imine) and poly(amido amine) containing terminal primary amines are characterized by generation and concentration-dependent toxicity [108]. Several surface modifications have been performed on dendrimers to enhance their specific targeting capability.

Polyethylene glycol modification increases the biocompatibility of dendrimers by prolonging the blood circulation time of dendrimers in vivo and also tumor accumulation through the enhanced permeability and retention (EPR) effect [41]. Kaminskas et al. modified the surface of dendrimers using PEG, which resulted in the reduced metabolic lability and increased uptake into the tumors [78]. Mehta et al. reported PEGylated polylysine dendrimers. The largest G5 PEG1100 dendrimers displayed good tumor uptake and retention. However, the drug release was slow, thereby limiting the anticancer activity of the formulation. The smallest G4 PEG570 dendrimer tumor uptake was limited, and the intermediate-sized dendrimer displayed significant tumor uptake and good retention. The PEG molecular weight and the dendrimer size had a significant effect on the therapeutic efficacy of the dendrimer formulations [79]. PEG is also used as a drug spacer. Aleanizy et al. reported trastuzumab-grafted dendrimers. The drug was conjugated to the surface of dendrimer via maleimide-poly(ethylene) glycol-*N*-hydroxysuccinimide linker to promote internalization of the formulation via receptor-mediated endocytosis [88]. Zhang et al. reported PEGylated lysine peptide dendrimers loaded with gemcitabine via the enzyme-cleavable linker, glycyl phenylalanyl leucyl glycine tetra-peptide. The uptake of the drug into the tumor was high, with a tumor growth inhibition value of over 90% compared to gemcitabine [104]. Dendrimer was modified with PEG and loaded with gold nanoparticles for MR/CT molecular imaging of breast cancer cells. The uptake of the gold nanoparticles into the liver and kidney was low indicating the nanoparticles escaped the reticuloendothelial system in the liver. The uptake of the gold particle into the tumor was high promoting effective MR/CT imaging of the tumors. The formulation displayed prolonged blood circulation time attributed to the modification of PEG moieties on the dendrimer surface [94].

Dendrimers also suffer from other limitations, such as inadequate tumor accumulation and rapid systemic clearance [79,82,108]. PAMAM dendrimers (G2–G4) were reported to display rapid renal clearance, and some of them were taken up by the kidney [113]. The rapid elimination of some dendrimers from plasma circulation insufficient tumor accumulation [108]. The use of targeting moieties has been reported to be a unique approach to promote targeting drug delivery to the tumor [75,84,105]. A variability of therapeutics outcomes from animal studies results from the diversity of animal models used, different duration of treatment, etc. Some strains of animal models react differently to the tested formulations, and some of the animals may have the capability to display adaptation that can withstand the toxic side effects of the tested formulations. The use of different strains of animals influences the data interpretation, toxicity profiles, etc. [110].

## 5. Polymeric Micelles

**Figure 3:**
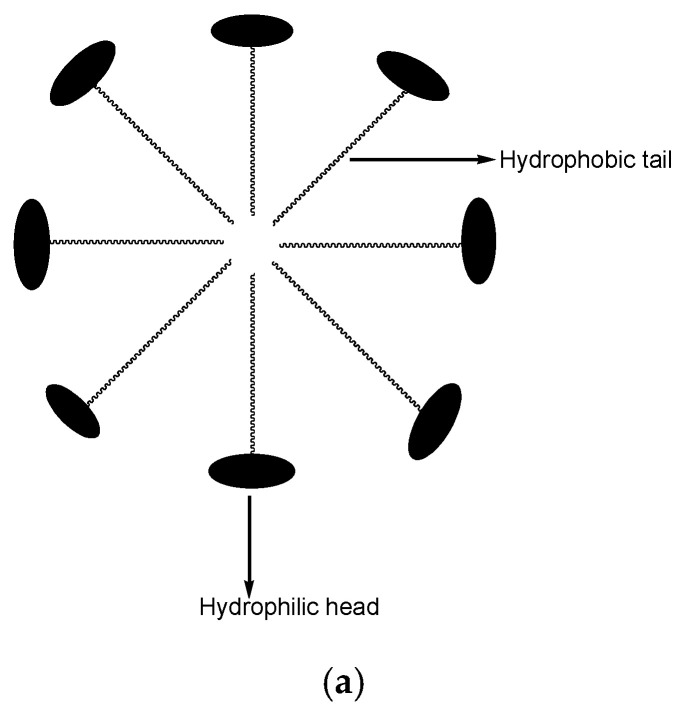

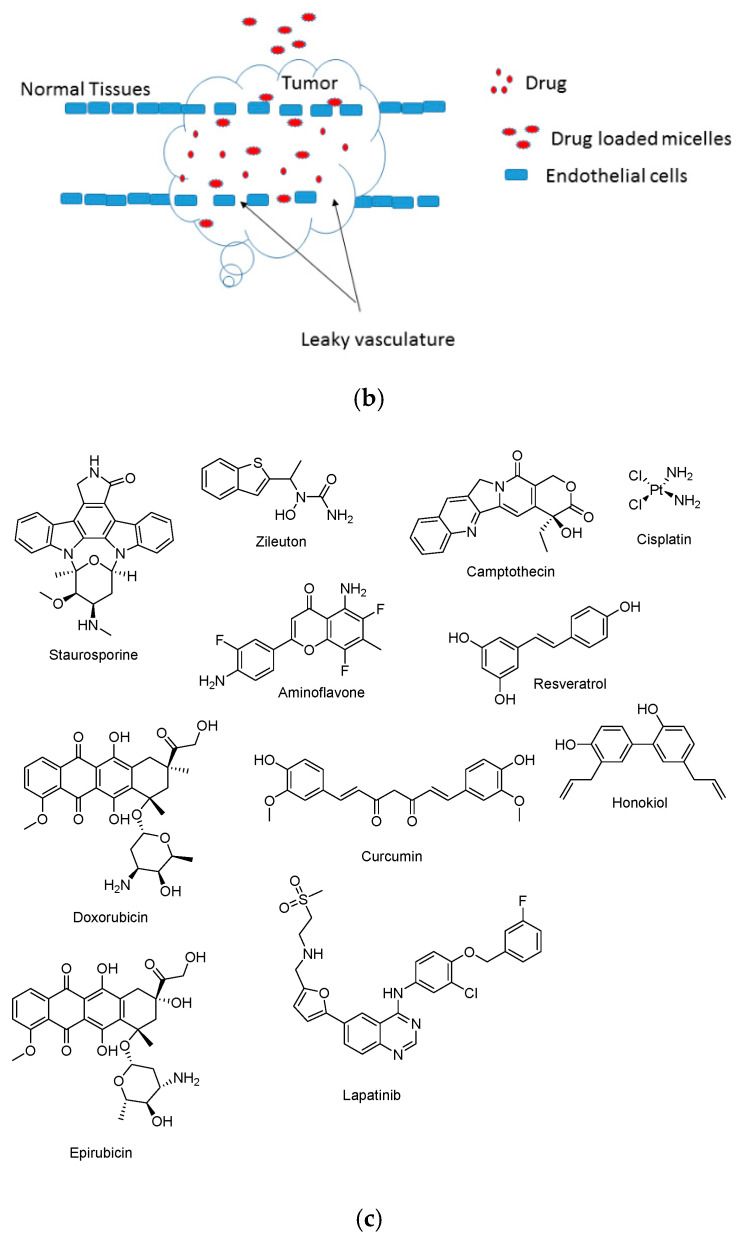

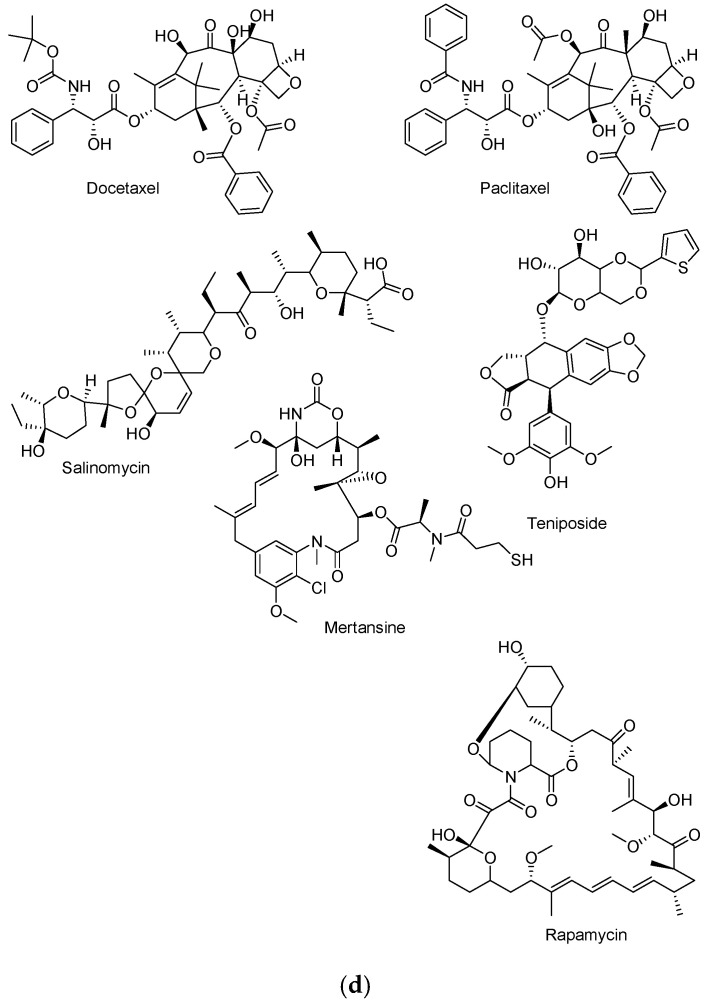
(**a**): Schematic diagram of polymeric micelles. (**b**): A schematic diagram illustrating the uptake of micelles formulation into tumor cells. (**c**): Structures of some anticancer drugs loaded into micelles. (**d**): Structures of some anticancer drugs loaded into micelles.

Micelles are self-assembled or colloidal nanoparticles/nanocarriers with a mean particle size ranging from 5 to 100 nm [114,115]. They consist of surfactants or amphiphiles and are composed of two different parts: hydrophobic tails and a hydrophilic head (Figure 3a) [116,117]. The concentration whereby the micelles are produced is called critical micelle concentration [118]. Several factors affect the production of the micelles, such as temperature, the solvent used, size of the hydrophobic domain in the amphiphilic molecule, and the concentration of amphiphiles [116]. The advantages of micelles in drug delivery include high drug loading capacity, high drug encapsulation efficiency, high drug cellular uptake due to the micellar nanosize (Figure 3b), improved drug stability, and they are easily eliminated from the biological environment after biodegradation, protect normal body cells from drug toxicity, useful for combination therapy, and they improve pharmacokinetic parameters of encapsulated drugs [119,120,121]. Different anticancer drugs have been loaded into micelles resulting inan improved anticancer activity of the loaded drugs in vitro and in vivo (Figure 3c,d) (Table 4).

### 5.1. Polymeric Micelles Loaded with Docetaxel

Loading docetaxel into micelles has been reported by several researchers to result in good therapeutic outcomes in vitro and in vivo when compared to the free drug, docetaxel (Table 4). It has been designed for combination therapy and sustained release profile of both loaded drugs. Guo et al. prepared methoxylpoly(ethylene glycol)-poly(d,l-lactide) copolymer (mPEG-PDLA)-based micelles co-loaded with docetaxel and resveratrol at a 1:1 fixed ratio for drug-resistant breast tumor therapy [122]. The average particle size and PDI of the mPEG-PDLA micelles were 17.1 ± 3.2 nm and 0.27 ± 0.01, respectively. The % drug loading of docetaxel and resveratrol in the polymeric micelles was 16.87 and 16.89%, respectively. The TEM results showed spherical morphology, which was uniform, and the particle size was in the range of 20–50 nm. The in vitro drug release profiles were fast for both drugs in the first 12 h at physiological pH of 7.4. After 12 h, the drug release mechanism was slow, and the cumulative release of both drugs was almost 80% in 3 days. Combining both drugs at a ratio of 1:1 (*w*/*w*) resulted in a significant synergistic effect against the MCF-7 cells. The loading of both drugs in the micelles resulted in prolonged drug release profiles and improved cytotoxicity in vitro [122]. The cytotoxicity assessment in vitro of the blank mPEG-PDLA-based micelles in MCF-7 breast tumor cells using MTT assay showed a non-cytotoxic effect indicating their safety and biocompatibility of the micelles. The co-loaded polymeric micelles demonstrated the highest cytotoxic efficacy that resulted in 70% of the MCF-7 cell death, which was greater than the activity of micelles loaded with the same concentration of either docetaxel or resveratrol. Furthermore, the pharmacokinetic study in vivo of co-loaded polymeric micelles demonstrated that these polymeric micelles mostly prolonged the exposure period of docetaxel and resveratrol in the blood plasma circulation. The AUC_(0→*t*)_ value of the micelles loaded with docetaxel was 3.0-fold higher than the free drug in vivo after intravenous administration [122]. Lang et al. formulated tumor-environment-responsive micelles entrapped with docetaxel for metastatic breast tumor treatment [123]. These micelles were prepared from amphiphilic copolymer, poly((1,4-butanediol)-diacrylate-*b*-N,N-diisopropylethylenediamine)-peptide-polyethylene glycol (PEG) (BD-peptide-PEG), matrix metallo-proteinase (MMP)-responsive polymer, and poly((1,4-butanediol)-diacrylate-*b*-N,N-diisopropyl-ethylenediamine)-polyethyleneimine (BD-PEI). The drug encapsulation efficiency and drug loading of the micelles were 80.54% and 9.81%, respectively. The average particle size was 24.36 nm, with a surface charge of −1.44 mV. The cellular uptake assessment of micelles displayed higher cellular uptake in the 4T1 breast cancer model in vivo than the cell culture medium in vitro. The polymeric micelles were cytotoxic in 4T1 breast cancer cells, indicating that polymeric micelles are specific and important as a drug delivery system. The drug release from the micelles formulation in acidic endo/lysosomes is via the dissociation of the micelle resulting from the protonation of the hydrophobic block. The drug-loaded micelle inhibited primary tumor growth and pulmonary metastasis effectively with a prolonged circulation time. Its efficient uptake into tumor cells is a promising approach for treating metastatic breast cancer [123].

Drug-loaded micelles can be designed to be dose-dependent. Logie et al. evaluated taxane-binding peptide-modified micelles encapsulated with docetaxel employing poly(d,l-lactide-*co*-2-methyl-2-carboxytrimethylene carbonate) [124]. The average particle size of the micelle was 121 ± 25 nm, with a distribution of 0.15 ± 0.03. The pharmacokinetic profiles of the free drug and the polymeric micelles showed a significant reduction in the concentration of plasma immediately after administration, and displayed modest enhancement of the parameters of pharmacokinetic for 7 h. The in vivo cytotoxicity studies of docetaxel loaded micelles against MDA-MB-231/H2N breast tumor model in mice showed a 72% tumor growth inhibition at a higher dose (8 mg/kg), while at a lower dose (5 mg/kg), a 50% tumor growth inhibition was visible which is the same as the free drug in a lower dose [124]. The concentration of the formulation used in vivo influenced their cytotoxic effects. Hu et al. formulated micelles from methacrylated block copolymer holding monomethoxy PEG encapsulated with docetaxel [125]. The in vitro drug release kinetics showed a sustained docetaxel release from these polymeric micelles at physiological environments (pH 7.4), due to the covalent conjugation via a hydrolyzable linker. Furthermore, the in vivo antitumor study in mice bearing MDA-MB-231 breast cancer xenografts showed potent chemotherapeutic activity compared to free docetaxel (Taxotere) [125]. The nature of the linker used for the conjugation influenced the drug release profile of the formulation. A sustained release profile is suitable for drug delivery systems to overcome drug toxicity.

Li and co-workers formulated Lyp-1 modified PEI derivative-PCL-*g*-PEI-based micelles co-loaded with docetaxel and near infrared (NIR) dye (IR820) for breast cancer chemotherapy [126]. The average particle size was 38 nm with a surface charge of +5 mV. The in vitro drug release profile indicated that docetaxel and IR820 were released slowly from the micelles, when compared to the free drugs, docetaxel and 1R820, respectively. The Lyp-1 modified dual drug-loaded micelles demonstrated high growth inhibition of 4T1 breast tumor cells when compared to the free drug and unmodified micelles [126]. Kutty and Feng prepared d-α-cetuximab-conjugated tocopheryl polyethylene glycol succinate (TPGS)-based micelles loaded with docetaxel for the treatment of triple-negative breast cancer (TNBC) therapy. The cytotoxicity analysis demonstrated that the anticancer effects of docetaxel could be greatly improved by incorporating it into the cetuximab-conjugated TPGS micelles. The cytotoxic effect of the formulation was 223.8 and 205.6-fold higher than the control (Taxotere^®^) on the MDA-MB-231 and MDA-MB-468 breast cancer cell lines, respectively revealing the efficacy of the micelles [127].

Drug-loaded micelles display enhanced oral drug availability. Wang et al. formulated PEG–PCL-based micelle hydrogels for oral drug delivery of docetaxel for breast cancer treatment. The particle size was 20 nm with approximately 7.76% drug loading capacity, which enhanced docetaxel absorption in the intestine tract. The pharmacokinetic analysis showed that docetaxel-loaded micelle-hydrogel significantly enhanced the oral drug bioavailability by 10-fold when compared to docetaxel-loaded micelles. The cytotoxicity analysis demonstrated that the docetaxel-micelle hydrogels were effective in inhibiting the tumor growth in 4T1 breast cancer model, and decreased systemic toxicity [128]. Micelles have been reported to exhibit significant tumor growth. Tan et al. formulated and evaluated mPEG-polyester micelles loaded with docetaxel for breast cancer treatment. The concentration of docetaxel-loaded mPEG-polyester micelles uptake in the tumor tissue and plasma was high when compared to the free docetaxel. In addition, the docetaxel entrapped micelles demonstrated higher tumor growth inhibition when compared to the free docetaxel in vitro and in vivo [129].

Drug-loaded micelles are effective in suppressing breast cancer metastasis. Li et al. formulated small-sized mPEG_2000_-*b*-PDLLA_1300_ micelles incorporated with docetaxel for breast cancer metastasis suppression. The cytotoxicity analysis demonstrated that the drug loaded micelles revealed similar activity as the free drug, docetaxel in cellular growth suppression of the primary tumors with a significant anticancer activity in 4T1 mouse breast tumor metastasis model [130]. Raza et al. synthesized Dextran-PLGA-incorporated docetaxel micelles for the treatment of breast cancer. The micelles average particle size was 96.5 nm, with a drug encapsulation of 54.85%. The in vitro cytotoxicity experiment showed that the anticancer activity of docetaxel incorporated in the micelles against MDA-MB-231 and MCF-7 cell lines was improved by 100%. The pharmacokinetic profile of the formulation revealed 16-fold enhanced bioavailability with a AUC_(0–∞)_ of 16367.39 μg·mL^−1·^h when compared to the free drug, which was 1206.75 μg·mL^−1^·h. The drug clearance of the formulation was reduced, revealing the extended residence of the drug in the biological system with the half-life of the drug increased by 5-fold, when compared to the free drug [131]. Kutty et al. formulated cetuximab conjugated TPGS micelles encapsulated with docetaxel for TNBC therapy. The ex vivo and in vivo cytotoxicity analysis of micelles showed the effective targeted and hindered EGFR-overexpressing on MDA-MB-231 TNBC breast tumor cell lines. The micelles accumulated in the tumours after administration via intravenous injection, and it was retained for 24 h. The uptake of the micelles was via receptor-mediated endocytosis. The tumours treated with the micelles exhibited improved cell cycle arrest and attenuated proliferation [132]. Koo et al. formulated docetaxel-loaded EG-PLys-PPhe micelles for breast cancer treatment. It was composed of a core-shell containing redox-responsive shell-specific cross-links and loaded with docetaxel [133]. The drug release was influenced by the concentration of glutathione, which resulted in a reductive cleavage of the disulfide cross-links in the shell domains. The in vivo tissue distribution and tumor accumulation of the formulation labeled with a near-infrared fluorescence (NIRF) dye, showed enhanced tumor-targeted ability of the formulation with prolonged stable circulation in the blood and enhanced permeation and retention (EPR) effect. The therapeutic efficacy of the formulation was enhanced in tumor-bearing mice when compared to the free drug [133]. Muthu et al. formulated TPGS micelles of transferrin co-loaded with docetaxel and ultra-bright gold nanoclusters. The in vitro anticancer studies demonstrated that these micelles were 71.73 times more effective when compared to the control (Taxotere^®^) after 24 h of incubation with the MDA-MB-231-luc breast cancer cells [134]. Tan et al. reported novel MPEG-PDLLA-PLL copolymer micelles for drug delivery of docetaxel to breast cancer cells. The in vitro and in vivo anticancer studies using MTT assay and mice model, respectively, demonstrated that these micelles were efficient in tumor cell growth inhibition against subcutaneous 4T1 and MCF-7 breast cancer cell lines [135].

Zhang and co-workers formulated PEG-*b*-PLGA copolymer micelles co-loaded with docetaxel and chloroquine for breast cancer targeting. The in vitro cytotoxicity analysis of docetaxel loaded PEG-*b*-PLGA micelles and dual drug-loaded PEG-*b*-PLGA micelles after 24 h of incubation with MCF-7 cancer cells showed an IC_50_ value of 22.30 ± 1.32 and 1.75 ± 0.43 mg/mL, respectively, which demonstrated a 12-fold more efficient treatment of the dual drug loaded micelles when compared to loading a single drug in the micelles formulation. Combining docetaxel with chloroquine in the micelle formulation significantly improved the cytotoxic effect [136]. Jun and co-workers prepared docetaxel loaded micelles utilizing tripodal cyclotriphosphazene amphiphilile [NP(PEG750) (GlyPheLeu)_2_Et]_3_ as a carrier. The in vivo studies on xenograft model demonstrated complete tumor suppression of the MDA-MB-231 breast cancer cells at a lower dose of 5 mg/kg compared to Taxotere^®^ [137]. Enteshari et al. prepared poly(styrenemaleic acid)-poly(amide-ether-ester-imide) co-polymeric nanomicelles encapsulated with docetaxel for breast cancer treatment. The results from these micelles showed considerably inhibited tumor growth of MC4-L2 breast tumors induced in BALB/c mice and increased animal survival when compared to the free docetaxel [138]. Varshosaz and co-workers formulated magnetic polyvinyl caprolactam–polyvinyl acetate–PEG micelles entrapped with docetaxel and Fe_3_O_4_ nanoparticles for breast cancer treatment **[139].** The average particle size was 144.3 nm, with a negative zeta potential of −2.58 mV and 70% drug-loading efficiency. The in vitro cytotoxicity analysis of docetaxel loaded polymeric using MTT assay displayed significantly more anticancer activity when compared to the free drug on MDA-MB-231 and MCF-7 breast cancer cells, but the blank magnetic polymeric micelles displayed no cytotoxic effect on the normal fibroblast cells [139].

Zheng and co-workers formulated polypeptide cationic micelles of PEG-PLL-PLLeu for co-delivery of docetaxel and siRNA-Bcl-2 for breast tumor therapy. The anticancer analysis demonstrated that the dual drug-loaded micelles revealed down-regulation of the anti-apoptotic Bcl-2 gene and improved antitumor efficacy with a smaller dose of docetaxel, resulting in the inhibition of tumor growth of MCF-7 breast cancer xenograft murine model when compared to the docetaxel and siRNA [140]. Tong et al. formulated phospholipid-based micelles loaded with docetaxel mPEG_2000_-distearoylphosphatidylethanolamine (DSPE) for the treatment of breast cancer. The micelles showed drug loading capacity of 3.14 ± 0.13% and encapsulation efficiency of 97.31 ± 2.95%. The cytotoxicity analysis demonstrated that docetaxel loaded micelles displayed similar antiproliferative efficacy as the control, (Taxotere^®^) in vitro. The formulation displayed good antitumor activity when compared to Taxotere^®^ in vivo against MCF-7 breast tumor induced nude mice, which can be attributed to the passive targeting of the cancer cells by the polymeric micelles [141].

Docetaxel is used either alone or in combination with other anticancer drugs for the treatment of cancers such as breast, ovarian, etc. [142]. It has side effects such as neutropenia, fluid retention, gastrointestinal complications etc. [143]. The incorporation of docetaxel into formulations of micelles improved its therapeutic efficacy. The micelles loaded with docetaxel displayed particle sizes in the range of 17.1–121 nm. The formulation displayed sustained drug release profile [122], improved bioavailability after intravenous administration [122,131], prolonged circulation time [123], inhibited tumor growth at high dose [121], enhanced oral drug bioavailability [127], tumor targeted capability [133,138], displayed similar effect with a clinically approved drug [141]. Docetaxel has been combined with some anticancer drugs in micelles formulation such as resveratrol [122], and chloroquine [136] for breast cancer treatment.

### 5.2. Polymeric Micelles Loaded with Doxorubicin 

Doxorubicin is used either alone or in combination with other anticancer drugs for the treatment of cancers such as bladder, breast, ovarian, etc. [144]. It exhibits low oral bioavailability, permeability, and acute toxicity to normal tissue. It has side effects such as cardiotoxicity that is not reversible and nephrotoxicity [145]. The incorporation of doxorubicin into formulations of micelles improved its therapeutic efficacy (Table 4).

Micelles loaded with DOX that displayed a pH-dependent drug release profile. Gao and co-workers formulated zwitterionic pH-responsive polymeric micelles for drug delivery of doxorubicin using hyaluronic acid (HA) as a nanocarrier [146]. The drug loading capacity and encapsulation efficiency of the HA micelles were 84.3% and 68.9%, respectively. The drug release mechanisms in vitro of the micelle loaded with doxorubicin were evaluated at pH 7.4 (physiological environment) and pH 5.0 (lysosome of cancer cells). The doxorubicin release from the micelle was slow, and it was less than 30% after 24 h at pH 7.4 while it was quick at pH 5.0 before 12 h and was 70% after 24 h. The in vitro anticancer studies of the drug-loaded polymeric micelles and individual drug against MCF-7 breast tumor cells utilizing a CCK-8 assay demonstrated that doxorubicin-loaded micelles exhibited higher growth inhibition (IC_50_ value in the range of 1.85–1.97 µg/mL) when compared to the free doxorubicin (IC_50_ value of 2.33 µg/mL). The cellular uptake assessment showed a successful uptake of the micelle formulation in the MCF-7 cells. Furthermore, in vivo anticancer studies showed that the drug-loaded micelles displayed significant tumor growth inhibition of approximately 80% when compared to the NaCl group (used as control) [146]. The formulation quick release in acidic pH inhibited the growth of the tumor and improved the cytotoxic effect.

Gao et al. formulated polymeric micelles using two polymers: poly(l-histidine) (PHis) (Mn 4700)-*b*-PEG (Mn 2000) and poly(l-lactide) (PLLA)(Mn 3000)-*b*-PEG(Mn 2000)-folate for the delivery of doxorubicin [147]. In vivo studies showed the absence of tumor metastasis in the lung and heart after treatment with the micelle formulation. The micelles suppressed the proliferation of cancer cells and inhibited tumor growth and cancer cell metastasis. Using folic acid as a targeting moiety combined with the pH-sensitive core phase carrier promoted targeted drug delivery and triggered drug release at the tumor sites. The pH-sensitive folate conjugated micelle loaded with DOX is a promising approach for the treatment of cancer metastasis. The encapsulation of DOX in the core of the micelle also protected the normal tissues from being exposed to the side effects of DOX [147]. The cytotoxicity of free DOX was higher than that of DOX carried by PLLA-*b*-PEG and PHis-*b*-PEG micelles. The cytotoxicity of DOX-loaded PHis-*b*-PEG was higher than DOX-loaded PLLA-*b*-PEG micelle because pH-dependent drug release from pH-sensitive PHis-*b*-PEG micelle at pH 6.8 when compared to the pH-insensitive PLLA-*b*-PEG micelle. The blank micelles did not display any cytotoxic effect on 4T1 cells revealing the biocompatible nature of the micelles.

Zhao et al. formulated micelles entrapped with an antitumor antibiotic, doxorubicin, for the eradication of cancer stem cells in TNBC utilizing pluronic block copolymers [141]. The antitumor effects of the free drug and polymeric micelles were evaluated against cancer stem cells in TNBC in vitro. The cytotoxicity efficacy of the drug-loaded micelles against MDA-MB-231 and MDA-MB-468 and potency in reducing the development of a colony of cancer stem cells when compared to free doxorubicin was studied. Furthermore, polymeric micelles were potent in tumor growth inhibition in vivo in the orthotopic tumor models formed from cancer stem cells, confirming polymeric micelles as potential therapeutics against TNBC [148]. Bae et al. prepared micelles for co-delivery of doxorubicin and phosphatidylinositol-3 kinase inhibitor wortmannin using PEG-*b*-poly(aspartate hydrazide) copolymers as a polymeric nanocarriers [149]. The particle size of the polymeric micelles employing DLS was less than 100 nm, which is preferable for tumor-specific drug delivery in vivo. The cytotoxicity evaluation in vitro against MCF-7 cells demonstrated that the anticancer efficacy was time- and dose-dependent and the cell viability was effectively suppressed after 3 days of incubation by dual drug-loaded polymeric micelles when compared to individual drug-loaded polymeric micelles and the free drugs [149]. Zhang and co-workers formulated crosslinked glutathione-sensitive carboxymethyl chitosan micelles co-loaded with doxorubicin and cisplatin for breast tumor therapy. The in vitro drug release profile demonstrated that these micelles were highly glutathione-sensitive. The cytotoxicity experiments showed that the dual drug-loaded micelles displayed high synergistic chemotherapeutic efficacy against HeLa cancer cells when compared to doxorubicin-loaded micelles, free doxorubicn, and plain polymeric micelles [150]. Varshosaz et al. formulated magnetic folate-dextran-retinoic acid micelles encapsulated with doxorubicin. The in vitro cytotoxicity analysis of doxorubicin-loaded polymeric micelles employing MTT assay showed good anticancer activity against MDA-MB-468 and MCF-7 breast tumor cells [151].

Lv et al. prepared amphiphilic PEG_2k_-PLA_5k_ micelles loaded with a combination of doxorubicin and curcumin to treat MDR breast cancer. The dual drug-loaded micelles displayed low efflux rate of doxorubicin, high cellular uptake, and high down-regulation of P-glycoprotein and inhibition of ATP activity. The co-loaded micelles also displayed high tumor accumulation and inhibitory effect on the tumor growth in xenograft model of drug-resistant MCF-7/ADR cells when compared to the free drugs revealing the efficacy of micelles loaded with two drugs [152]. Cuong and co-workers synthesized doxorubicin-loaded PEG-PCL-PEG micelles loaded with doxorubicin for breast cancer therapy. The circulation time of doxorubicin-loaded polymeric micelles in the plasma was prolonged when compared to the free doxorubicin in vivo. The tumor growth of MCF-7 breast cancer cells in nude mice was suppressed by multiple doses of the drug-loaded micelles when compared to multiple doses of free doxorubicin [153]. Yu and co-worker formulated amphiphilic diblock polymeric micelles loaded with doxorubicin to reverse doxorubicin resistance in breast cancer. The in vivo cytotoxicity analysis of the polymeric micelles demonstrated significant growth inhibition of doxorubicin-resistant MCF-7/ADR breast cancer in an orthotopic tumor-bearing mouse model [154].

Lee and co-workers reported folate modified PLLA/PEG polymeric micelles loaded with doxorubicin for resistant breast cancer therapy. The micelles displayed more than 90% anticancer efficacy in doxorubicin resistant MCF-7/DOX^R^ breast cancer cells. Furthermore, the in vivo antitumor experiments in the MCF-7/DOX^R^ xenograft model showed that the accumulated doxorubicin level of modified micelles in solid tumors was 20 times higher than free drug and 3 times higher than the unmodified polymeric micelles group [155]. Varshosaz and co-workers synthesized Pluronic^®^ F127-poly (methyl vinyl ether-alt-maleic acid) copolymer-based micelles entrapped with doxorubicin for breast cancer targeting [156]. The in vitro drug release profile at pH 5.5 and pH 7.4 after 4 h was sustained. However, doxorubicin release mechanism from the micelles was faster in pH 5.5 when compared to pH 7.4 demonstrating that after the cellular internalization of drug-loaded micelles into the cytosol of cancer cells, the pH of lysosomes can stimulate the fast doxorubicin release from the micelles. The in vitro cytotoxicity analysis demonstrated that the micelles loaded with doxorubicin destroyed approximately 48.9 ± 1.7% of MCF-7 breast tumor cells when compared to the free doxorubicin which destroyed 36.4 ± 1.1% of these breast tumor cells [156].

Sun et al. synthesized doxorubicin loaded PAA-*g*-PEG graft micelles for the treatment of breast carcinoma [157]. The in vivo cytotoxicity analysis of the drug-loaded micelles in 4T1 tumor induced nude mice breast carcinoma subcutaneous model demonstrated high accumulation of the formulation in the tumor than the free doxorubicin with a reduced distribution to important tissues. The anticancer effect of the polymeric micelles was importantly better when compared to the free doxorubicin, as confirmed by the tumor volume and body weight changes of the tumor-bearing mice [157]. Shuai et al. formulated micelles based on block copolymers of poly (ε-caprolactone) and PEG for the delivery of doxorubicin. Hemolytic experiments displayed that the free doxorubicin caused approximately 11% hemolysis at 200 µm/mL while no hemolysis was observed with the doxorubicin-loaded micelles at the same doxorubicin concentration of 200 µm/mL. The in vitro cytotoxicity study demonstrated that the micelles displayed a time-delayed anticancer activity in MCF-7 breast cancer cells because cancer cell viability was approximately 80% on 4th day and it drastically decreased on the 5th day at a concentration range between 0.01–10 µM [158]. Cuong and co-workers formulated folate-modified star-shaped PEG–PCL micelles loaded with doxorubicin for human breast cancer targeting. The cellular uptake analysis showed that the uptake of decorated micelle incorporated with doxorubicin was higher than the free drug in human MCF-7 breast tumor cells in a time-dependent manner. Furthermore, the experiment revealed that these folate-decorated star-shaped PEG–PCL micelles were non-toxic and are potential nanocarriers for cancer therapy [159]. Another study by Cuong and co-workers demonstrated that doxorubicin-incorporated micelles of a star-shaped poly(ε-caprolactone)-polyphosphoester block co-polymer significantly improved antitumor efficacy in MCF-7/drug-resistant breast cancer cells and MCF-7/drug-sensitive breast cancer cells after incubation in vitro [160].

Zhou et al. formulated micelles entrapped with doxorubicin from dextran and indomethacin for breast cancer treatment. These micelles demonstrated uniform size distribution with a mean diameter of 50 nm. The in vivo studies on male BALB/c nude mice bearing resistant MCF-7 breast xenograft tumor cells showed that the micelles significantly inhibited the tumor growth when compared to the control (saline) and doxorubicin [161]. Liao et al. prepared PEG–PCL cetuximab-immunomicelles encapsulated with doxorubicin and superparamagnetic iron oxide for EGFR-overexpressing breast tumor cell targeting. The in vitro anticancer analysis utilizing a MTT assay showed that the A431 breast cancer cells are highly sensitive to drug-loaded micelles when compared to MDA-MB-453 breast tumor cells. Furthermore, EGFR-overexpressing A431 cells, immunomicelles exhibited significant inhibitory effects on cell growth when the doxorubicin concentration was more than 2 µg/mL [162]. Chen et al. formulated micellar doxorubicin nanoparticles of mPEG-PCL-graft-cellulose to overcome MDR breast cancer cells. The flow cytometry and confocal laser scanning microscopy (CLSM) analysis in MCF7/ADR cells demonstrated more efficient endocytosis of the micellar doxorubicin nanoparticles in these cancer cells when compared to the diffusion of the free doxorubicin into the cytoplasm of the cancer cells. These results revealed that the doxorubicin loaded mPEG-PCL-*g*-cellulose micelles were effective in overcoming P-glycoprotein efflux in MDR breast tumor cells [163].

Zeng and co-workers prepared hyperbranched block poly-2,2-bis(methylol) propionic acid (bis-MPA)-PEG micelles for the delivery of doxorubicin in breast tumor cells. The particle size analysis of micelles using DLS and TEM showed an average hydrodynamic diameter in the range of 90–130 nm. The in vitro drug release profile from the drug-loaded polymeric micelles at physiological conditions was a biphasic release mechanism, with an initial slight burst drug release followed by a sustained release. The cytotoxicity analysis using MTT assay demonstrated that the anticancer efficacy of micelles was concentration dependent because the cell viability of MCF-7 and MDA-MB-468 breast cancer cells was decreased as the concentration of doxorubicin-loaded micelles increased [164]. Cheng et al. formulated amphiphilic poly(Îµ-caprolactone) micellar nanoparticles loaded with doxorubicin for breast cancer therapy. The in vitro cytotoxicity of the drug loaded micelles was higher against MCF-7 human breast cancer cells when compared to the plain micelles when incubated at a temperature increased above the LCST [165].

Danhier et al. formulated vitamin E-based tocopherol succinate (TOS)-TPGS micelles encapsulated with doxorubicin for breast tumor treatment [166]. The mean particle size of 78 nm with a negative surface charge of −7 mV. The in vitro cytotoxicity evaluation of doxorubicin-loaded micelles showed that they exhibited higher anticancer activity when compared to the free doxorubicin against MCF-7 breast tumor cells after 24 h. Furthermore, the in vivo anticancer analysis revealed a 100% long-time survival of MCF-7 and CT26 induced mice treated with the micelles loaded with doxorubicin when compared to the free drug [166]. Liu and co-workers synthesized mPEG-PLA micellar nanoparticles co-loaded with doxorubicin and gemcitabine for synergistic anticancer efficacy on breast cancer cells. The in vitro cytotoxicity analysis of the co-loaded micelles using MTT assay exhibited significant synergistic anticancer effect against MCF-7 breast cancer cells [167]. Cagel et al. formulated micelles from Tetronic T1107, Pluronic F127, TPGS, and incorporated doxorubicin resulting in improved cytotoxicity on breast cancer cell lines. The in vitro drug release was significantly high at an acidic tumor microenvironment of pH 5.5 when compared to the physiological environment of pH 7.4. The in vitro cytotoxicity analysis of the micelles revealed higher anticancer efficacy against TNBC cells (MDA-MB- 231) when compared to the control (Doxil^®^) [168].

Chen and co-workers formulated Pluronic-based functional micelles co-loaded with doxorubicin and paclitaxel for MDR breast cancer treatment [169]. The in vitro drug release profile was sustained for both drugs. The co-loaded micelles significantly reduced the cell viability of MCF-7 cancer cells when compared to a single drug-loaded micelles. Furthermore, the in vivo antitumor analysis revealed high anticancer activity in MCF-7/ADR tumor-bearing mice for the dual drug-loaded micelles when compared to the combined administration of doxorubicin and paclitaxel [169]. Wang et al. designed MPEG-PCL-4-formylbenzoic acid (FBA) micellar nanoparticles for combination therapy of paclitaxel and doxorubicin. The combination resulted in synergistic antitumor efficacy against MCF-7 breast cancer cells when compared to the single drug-loaded micelles and the free drugs [170].

### 5.3. Polymeric Micelles Loaded with Paclitaxel 

Paclitaxel is used for the treatment of various types of cancers, including breast cancer. The resistance of breast cancer to paclitaxel is due to certain genes, ABC transporters, etc. [171,172]. Some of the side effects of paclitaxel include peripheral neuropathy, poor solubility, etc. which are responsible for more research on its use in several preclinical and clinical studies [171]. The incorporation of paclitaxel into micelles have been reported to improve its therapeutic outcomes in vitro and in vivo (Table 4).

Wang et al. designed and evaluated carboxymethyl chitosan-rhein polymeric micelles for oral delivery of paclitaxel [173]. The entrapment efficiency and drug loading of paclitaxel in chitosan-based micelles were 86.99 ± 12.26% and 35.24 ± 1.58%, respectively. The drug-loaded polymeric micelles improved the paclitaxel bioavailability. The drug release profile of polymeric micelles was sustained with a slowed drug release of 66.14% in 2 days at physiological conditions in vitro. The in vitro synergistic anticancer studies against the cancer cells between paclitaxel and chitosan-rhein conjugates was assessed utilizing MCF-7 cancer cells. Cell viability outcomes demonstrated a dose-dependent toxicity of paclitaxel. In addition, the low cytotoxic effect of the polymeric micelles was observed at experimental doses. The IC_50_ value of paclitaxel-loaded polymeric micelles and free paclitaxel in MCF-7 cells was 17.62 nM and 30.70 nM for 3 days, respectively [173]. The sustained release profile of the formulation has the potential to overcome drug resistance. Zajdel et al. prepared polylactide-*co*-poly(ethylene glycol) micelles loaded with a combination of paclitaxel and lapatinib. The micelles diameter was 20 nm in diameter and spherical morphology. The biodistribution, cellular uptake, circulation time and the efficacy of the micelles was influenced by the morphology. Over 8.0% and 8.7% of paclitaxel and lapatinib was released from the micelles after 1 h. After 24 h, the release of paclitaxel was 61.2% while the release of lapatinib was 27.6%. The high release of paclitaxel is attributed to factors such as diffusion, higher molar mass and higher loading content compared to lapatinib. The micellar system passively targeted the cancer cells by enhanced permeability and retention effect. The drug-loaded micelles displayed enhanced cytotoxic effects compared to the free drugs after 48 h and 72 h on MCF-7 cell lines. The drug combination using the micelles showed synergistic effects by reducing the viability of HER-2 negative breast cancer cell lines [174]. Wu and co-workers prepared and evaluated folate targeted biodegradable polymeric MPEG-*b*-P(LA-*co*-DHC/FA) micelles loaded with paclitaxel for breast cancer cell targeting [175]. The in vivo antitumor results in EMT-6 breast cancer cell line showed that the mean tumor masses were 0.18, 0.33, and 0.49 g for the drug-loaded polymeric micelles, free paclitaxel, and saline (control), respectively, indicating the potent anticancer efficacy of micelles when compared to free drug and control. Furthermore, the tumor growth inhibition of the three formulation groups was 66.1, 36.6, and 1.0%, respectively [168].

Wang et al. formulated pH-sensitive micelles using poly(2-ethyl-2-oxazoline)-poly(d,l-lactide) for the combination of paclitaxel and honokiol. The mean particle size was in the range of 41–44 nm with PDI that is less than 0.3, indicating the good stability of the micelles and their potential capability to deliver drugs to the tumor. TEM image of polymeric micelles was sphere-shaped with uniform particle size. The drug release profile from the formulation was fast followed by a slow and sustained drug release of both loaded drugs for 2 days at pH 5.4 and 7.4. The in vitro antitumor studies of the polymeric micelles were studied in the MCF-7/ADR breast cancer line employing SRB assay. The anticancer results showed that the cell viability was in the range of 99.5 ± 11.5%–100.0 ± 7.92% at a polymeric micelle concentration of 14.31 µg/mL, followed by a rapid decrease [176].

Oda et al. designed lyophilized diethylenetriaminepentaacetic acid-functionalized micelles encapsulated with paclitaxel utilizing 1,2-distearoyl-sn-glycero-3-phosphoethanolamine-N [methoxy(polyethyleneglycol)-2000] as a polymer. The lyophilization route did not alter the biological and physicochemical properties of polymeric micelles. Furthermore, the biodistribution profile of polymeric micelles displayed high uptake in the spleen, liver, and kidney in mice-bearing 4T1 tumor, indicating that polymeric micelles can be easily eliminated via these routes [177]. Zhang et al. formulated and evaluated polymeric micelles entrapped with paclitaxel that is based on poly(ɛ-caprolactone)-poly(ethylene glycol)-poly(ɛ-caprolactone) triblock copolymers [178]. The anticancer outcomes of drug-loaded polymeric micelles in the EMT6 breast tumor model showed that the cell viability was lower at 3 days by 2–10 times when compared to day 1, indicating the drug capability to kill tumor cells effectively and it also revealed that there no need for a prolonged exposure period [178].

Zhang and co-workers formulated octreotide modified PEG-*b*-PCL-based micelles encapsulated with paclitaxel or salinomycin for breast tumor therapy. The average particle size of the polymeric micelles ranged between 25 and 30 nm with an encapsulation efficiency of more than 90%. The in vitro cytotoxicity analysis of dual drug-loaded micelles demonstrated stronger growth inhibition against MCF-7 breast cancer cells when compared to the free drugs and the single drug-loaded micelles [179]. Yin and co-workers formulated hyaluronic acid-based amphiphilic micelles co-loaded with hydrophobic paclitaxel and hydrophilic AURKA specific siRNA for breast cancer treatment. The in vivo anticancer analysis using BalB/c nude mice bearing MDA-MB- 231 tumors demonstrated that the volume of the tumors in mice that received polymeric micelles encapsulated with paclitaxel or si-AURKA specific siRNA were notably smaller than the mice treated with the controls (saline and Taxol^®^). Furthermore, the mice treated with co-loaded micelles showed the synergistic antitumor effect of the loaded drugs [180]. Wang et al. formulated PEG-PDLLA micelles loaded with paclitaxel for breast cancer therapy. The in vivo imaging demonstrated that the polymeric micelles had outstanding specific cancer cells targeting effect and improved drug accumulation. The in vivo and in vitro antitumor experiments demonstrated that the paclitaxel-loaded micelles possessed high inhibition of the cancer cells and cell apoptosis on MDA-MB-231 breast cancer cells [181].

Kelishady and co-workers formulated Pluronic F127 polymeric micelles co-loaded with paclitaxel and lapatinib for the treatment of metastatic breast cancer [182]. The in vitro drug release profile of the micelles showed a burst release of 43% of PTX and 24% of lapatinib in the first 2 h, followed by a sustained drug release mechanism for 25 h. The in vitro anticancer analysis showed that the dual drug-loaded micelles significantly suppressed the proliferation of resistant T-47D breast cancer cell lines with IC_50_ value of 0.6 ± 0.1 µg/mL when compared to the free drug combination mixture of paclitaxel and lapatinib that showed an IC_50_ value of 6.7 ± 1.2 µg/mL [182]. Hou et al. formulated novel Soluplus^®^-Solutol^®^ HS15 binary mixed micelles loaded with paclitaxel. The in vivo anticancer experiments using male nude mice bearing MDA-MB-231 breast cancer cells demonstrated that these micelles achieved higher antitumor efficacy of 57.66% when compared to 41.13% of the free paclitaxel [183]. The hyaluronic acid-shelled acid-activatable paclitaxel micelles designed by Zhong and co-workers were effective in vivo against mice induced with MCF-7 human breast tumor with little adverse effects. The formulation completely inhibited the growth of the tumor with 100% survival rate over 55 days [184].

Hasenstein et al. formulated a PEG-PLA multidrug-loaded micelle called Triolimus containing paclitaxel, 17-AAG, and rapamycin. The drug-loaded micelle significantly inhibited MDA-MB-231 and 549 breast cancer tumor growth in vivo when compared to the micelles loaded with paclitaxel alone [185]. Lee et al. performed a phase II clinical trial of monomethoxy poly(ethylene glycol)-block-poly(d,l-lactide) micelle called Genexol-PM encapsulated with paclitaxel for metastatic breast cancer therapy in 41 patients. The results from this clinical studies demonstrated that 37 patients who were administered the formulation via intravenous infusion at a dosage of 300 mg/m^2^ for a period of three weeks as a first-line chemotherapy therapy for their metastatic breast cancer showed a response rate of 59.5% and two responses were observed in four breast cancer patients treated in the second-line setting for their metastatic cancer. Febrile neutropenia was not reported in any of the patients [186]. Mei and co-workers formulated α-conotoxin ImI modified PEG-DSPE micelles loaded with paclitaxel. The micelles displayed higher anticancer efficacy by inducing significant cell apoptosis and significant antitumor activity in MCF-7 tumor-bearing mice [187]. Liu et al. prepared PLGA-*g*-dextran micelles encapsulated with paclitaxel for breast cancer therapy. These micelles demonstrated higher anticancer efficacy against MCF-7 breast cancer cells than the control, (Taxol^®^) in vitro showing that paclitaxel loaded PLGA-*g*-dextran micelles can overcome the multidrug resistance in human breast carcinoma cells [188]. Yang et al. formulated micellar drug delivery systems that are based on poly(ethylene glycol)-benzoic imine-poly(g-benzyl-l-aspartate)-*b*-poly(1-vinylimidazole) block copolymer (PPBV) layer detachment for co-delivery of paclitaxel and curcumin. The in vivo cytotoxicity using the mice bearing subcutaneous MCF-7 breast tumors demonstrated that the average tumor volume after treatment with the dual drug-loaded PPBV micelles was 6.6, 5.4, 5.2, and 4.7-fold smaller when compared to those treated with 0.9% NaCl, a combination of paclitaxel and curcumin, PPBV micelles loaded with paclitaxel alone, and PPBV micelles loaded with curcumin alone, respectively [189]. Han et al. formulated and evaluated HA-based micelle conjugates loaded with paclitaxel for breast tumor therapy. The cellular uptake analysis revealed that the HA micelles loaded with paclitaxel could be specifically and efficiently internalized into 4T1 breast cancer cells via endocytosis. In vitro cytotoxicity analysis showed that the paclitaxel loaded micelles enhanced the selectivity of paclitaxel for destroying the 4T1 cancer cells when compared to the free paclitaxel **[190]**.

Wang and co-workers formulated MCF-7 cell-specific phage protein modified PEG-phosphatidylethanolamine micelles loaded with paclitaxel for breast cancer targeting. The fluorescence microscopy demonstrated that MCF-7 targeted phage micelles were bound to the target cells, MCF-7 when compared to the non-target cells. The in vitro cytotoxicity analysis of the micelles loaded with paclitaxel exhibited a significant higher antitumor efficacy on MCF-7 breast cancer cells than the free paclitaxel [191]. Bernabeu et al. formulated mixed micelles that are based on two copolymers of polyvinyl caprolactam–polyvinyl acetate–PEG (Soluplus^®^) and TPGS for the incorporation of paclitaxel with improved anticancer activity on breast cancer cell lines. The in vitro cytotoxicity experiments showed that the mixed micelles loaded with paclitaxel demonstrated superior anticancer efficacy when compared to the free paclitaxel solution against MCF-7 and TNBC cells (MDA-MB-231) [192]. Chen et al. formulated poly (β-amino ester) copolymer micelles loaded with paclitaxel for breast cancer metastasis treatment. The in vivo and in vitro cytotoxicity studies of micelles showed that the micelles induced drug cellular uptake and significant MDA-MB-231 breast cancer cell disruption and suppressed breast tumor metastasis [193].

Wang and co-workers prepared PEG-PE–based micellar nanoparticles loaded with paclitaxel. The micellar nanoparticles demonstrated improved antitumor activity by promoting enhanced cell apoptosis against MCF-7 and reduced breast cancer cell proliferation [194]. Wang et al. also synthesized biodegradable mPEG-poly(caprolactone) micelles for co-delivery of paclitaxel and honokiol. The in vitro drug release profile was a sustained release of both drugs from the micelles. The in vitro cytotoxicity analysis and the cellular uptake studies of the co-loaded micelles showed an increased antitumor activity on the 4T1 breast cancer cells by promoting cell apoptosis and higher cellular uptake by the breast cancer cells [195]. Lang et al. formulated polymeric micelles loaded with paclitaxel for improved metastatic breast cancer therapy. The in vivo cytotoxicity studies of the micelles demonstrated 15-fold higher intratumor paclitaxel accumulation when compared to the commercially available paclitaxel, and achieved a growth tumor inhibition rate of 96.8% on the 4T1 metastatic breast cancer mice model [196]. Wang and workers reported folate modified pluronic copolymer micelles encapsulated with paclitaxel. Their anticancer activity on MDR MCF-7 ADR breast cancer cells was high when compared to paclitaxel solution [197]. The finding revealed the efficacy of using targeting moiety.

Lu et al. synthesized PEG-derivatized embelin as a nanomicellar drug delivery system loaded with paclitaxel for breast cancer therapy. The in vitro cell uptake of the micelles by the cancer cells were significantly high. The in vitro antitumor analysis demonstrated that paclitaxel loaded micelles revealed higher levels of antitumor effect on 4T1.2 breast cancer cells when compared to Taxol formulation [198]. Zhu et al. synthesized low-density lipoprotein–N-succinyl chitosan–cystamine–urocanic acid micelles for the co-delivery of paclitaxel and siRNA. The in vitro cellular uptake analysis demonstrated an initial significant accumulation of the co-loaded micelles in lyso/endosomes and a gradual diffusion into the entire cytoplasm of MCF-7 breast tumor cells. The in vivo cytotoxicity studies of micelles co-loaded with paclitaxel and siRNA using MCF-7 breast tumor-bearing nude mice showed the superior anticancer efficacy revealing a synergistic anticancer effect of combining paclitaxel and siRNA, when compared to the paclitaxel loaded micelles and siRNA loaded micelles [199].

### 5.4. Polymeric Micelles Loaded with Curcumin

The clinical use of curcumin remains a challenge resulting from its poor solubility, bioavailability, and stability. To improve its bioavailability and solubility, it is loaded into nanoparticles such as Chen et al. formulated phosphorylated calixarene-based micelles loaded with curcumin for TNBC treatment [200]. The DLS analysis of curcumin loaded micelles demonstrated a mean particle size of 3.86 ± 0.32 nm with a negative zeta potential of −25.18 ± 5.74 mV and PDI value of 0.125 ± 0.078, revealing a narrow size distribution. The encapsulation efficiency and drug loading capacity were 95.40 ± 4.50 and 17.10 ± 1.25%, respectively. The in vitro drug release profile was slow at pH 7.4 and fast at pH 5.5, indicating continuous sustained drug release pattern and prolonged release at neutral pH. The in vitro cytotoxicity studies using the MTT assay demonstrated concentration-dependent pattern against BT-549 breast cancer cells with IC_50_ value of 2.67 ± 0.40 µg/mL for curcumin loaded micelles, 9.78 ± 0.51 µg/mL for curcumin, and 194.35 ± 23.87 µg/mL for plain micelles. The similar anticancer trends were reported in MCF-7 breast tumor cells. Furthermore, the in vivo drug release studies using mice bearing BT-549 tumors demonstrated improved sustained curcumin release around the tumor mass at 24 h after intratumoral injection, revealing curcumin loaded micelles can potentially accumulate at the tumor environment. Loading curcumin into the micelles facilitated curcumin capability to hinder the nuclear activity of androgen receptor, induce cell cycle arrest and apoptosis. These mechanisms mentioned above contributed to the formulation ability to inhibit the growth of BT-549 tumor xenografts in mice, without causing any significant side effects during the 14 days of treatment [200].

Huang and co-workers formulated PEGylated micelles co-encapsulated with curcumin and doxorubicin, exerting a synergistic effect in MDR breast tumor cells. The micelles showed a mean particle size of 90.64 ± 0.15 nm and a negative surface charge of −15.3 ± 0.8 mV. The in vitro cytotoxicity of polymeric micelles was high with a IC_50_ value of 3.10 µg/mL when compared to free doxorubicin (IC_50_ of 31.99 µg/mL). Moreover, the micelles exhibited high cellular uptake and reduced cellular efflux. The combination index of the dual drug-loaded micelles was 0.17, which shows a strong synergistic anticancer activity against MCF-7/ADR breast cancer cells [201]. Jung and co-workers formulated epidermal growth factor-modified DSPE-PEG phospholipid micelle nanoparticles loaded with curcumin for breast cancer treatment. The micelle was conjugated with epidermal growth factor for specific targeting of epidermal growth factor receptors overexpressed on TNBC. The in vitro cytotoxicity study on MDA-MB-468 breast cancer cells revealed that the highest dose tested of 10 µM of free curcumin and curcumin loaded micelles significantly reduced cell viability to 41.5 ± 2.8 and 63.1 ± 8.3%, respectively [202]. Medel et al. prepared curcumin-bortezomib loaded mPEG-*b*-PLA-based miceller nanoparticles for synergistic anticancer efficacy. The in vitro cellular uptake analysis of the nanoparticles with an average particle of 100–150 nm size showed a maximum cellular uptake by the MDA-MB 231 and MCF-7 breast cancer cells after 3 h, which can result in potential anticancer effects [203].

Liu et al. formulated MPEG-PCL copolymer micelles loaded with curcumin for breast tumor therapy. The DLS analysis of curcumin loaded micelles demonstrated an average particle size of 28.2 ± 1.8 nm with PDI of 0.136 ± 0.050 and surface charge of −0.41 ± 0.25 mV. The TEM results exhibited spherical-shaped morphology in aqueous solution, and the results were consistent with that of particle size analysis. The drug loading capacity and encapsulation efficiency of curcumin loaded micelles were 14.84 ± 0.11% and 98.91 ± 0.70%, respectively. The drug-loaded micelles exhibited higher anticancer activity against 4T1 breast tumor cells in vitro when compared to free curcumin in a dose-dependent manner [204]. The formulation was effective in the inhibition of tumor growth, spontaneous pulmonary metastasis and extended the life span of the 4T1 breast tumor model.

### 5.5. Polymeric Micelles Loaded with Platinum Drugs

Platinum-based drugs cause apoptosis by penetrating into the nucleus of cancer cells resulting in the formation of adducts with DNA. However, their use is limited pharmacologically by toxicity and poor water solubility. Wan and co-workers formulated poly (2-oxazoline)-based micelles co-loaded with cisplatin and paclitaxel for breast tumor treatment [205]. The pharmacokinetic analysis showed that the dual drug-loaded polymeric micelles increased the plasma half-life of each drug when compared to the single drug loaded micelles. The in vivo cytotoxicity experiments of the dual drug-loaded micelles demonstrated the superior anticancer efficacy of the dual drug-loaded micelles when compared to single drug-loaded micelles or drug solutions against multidrug resistant breast cancer LCC-6-MDR orthotopic tumor models [205]. Ahmad et al. designed MPEG-block-Poly (L-glutamic acid-*co*-l-phenylalanine) micelle nanoparticles loaded with cisplatin for human breast cancer treatment. The in vitro drug release profile showed that 30% of cisplatin was released at physiological pH and 39% of the drug was released at acidic pH of the lysosome. The in vitro anticancer studies employing MTT viability assay displayed that the cell proliferation inhibition of polymeric micelle nanoparticle against ZR-75-30 human breast cancer cells was time- and dose-dependent. The micelle nanoparticles indicated more inhibition with low IC_50_ values on ZR-75-30 cell line when compared to the plain micelles and free drug. The formulation displayed high stability and extended blood circulation time [206].

Wang and co-workers formulated folate-modified mPEG-*b*-poly(l-lactide-*co*-2-methyl-2-carboxylpropylene carbonate-platinum (II) complex micelles. The particle size analysis of the micelles exhibited the average particle size that ranged between 100–200 nm. The drug molecules were loaded in the core part of the micelles, and the folic acid moiety was designed on the corona of the micelles for targeted drug delivery. Folic acid receptors are overexpressed on breast cancers making it a good targeting moiety. The cytotoxicity was evaluated using MTT assay which demonstrated very little antitumor effect for the plain polymeric micelles against MCF-7 breast cancer cells. The drug content from the micelle was significant at the tumor site. The micelles exhibited prolonged blood circulation when compared to the free drug [207].

### 5.6. Polymeric Micelles Loaded with Other Anticancer Drugs 

Other types of anticancer drugs have been loaded into prepared micelles which revealed the efficacy of micelles for the treatment of breast cancer (Table 4). Teniposide, a semisynthetic derivative of podophyllotoxin damages the DNA in the replication process with the induction of cellular apoptosis. However, it exhibits low water solubility and is administered as an injection. Some side effects are hypersensitivity reactions, tachycardia, etc. Its elimination is rapid, with a widespread distribution to normal organs and tumor tissue, thereby decreasing its therapeutic efficacy with increased side effects [208,209].

Chu and co-worker prepared and evaluated polymeric micelles encapsulated with teniposide that are based on monomethoxy-poly(ethylene glycol)-poly(e-caprolactone-*co*-d,l-lactide) (MPEG-PCLA) copolymers for breast cancer therapy [210]. The mean particle size was 29.6 ± 0.3 nm, with a negative surface charge of −0.980 mV. The encapsulation efficiency and drug loading were 92.63 ± 2.05% and 18.53 ± 0.41%, respectively. The in vitro drug release profile of the micelles at physiological condition (pH 7.4) was slowed and sustained with about 33% and 78% release of teniposide from the polymeric micelles in the first 8 h and 72 h, respectively. The in vitro anticancer assessment of the drug-loaded micelles using MCF-7 cancer cell lines and MTT assay for 2 days showed that the drug-loaded micelles significantly inhibited cell growth with (IC_50_ value = 3.248 µg/mL) when compared to the free drug (IC_50_ value = 5.342 µg/mL) in a dose-dependent manner. Furthermore, the in vivo evaluation using the MCF-7-bearing nude mice model showed smaller relative tumor volumes for those groups treated with teniposide-loaded polymeric micelles than those treated with individual teniposide [210]. The teniposide-loaded MPEG-PCLA micelles improved the water solubility, reduced the toxicity, and enhanced the antitumor activity of teniposide. However, there is a need for further studies.

Epirubicin administration have been reported to present high relapse rate resulting from the presence of a subpopulation of cancer cells, known as cancer stem-like cells. These cells are resistant to the currently used conventional therapies. Zhang et al. formulated polymeric micelles co-loaded with epirubicin and staurosporine that are based on poly(ethylene glycol)-*b*-poly(aspartate-hydrazide-epirubicin) copolymer for the treatment of breast cancer. The cytotoxicity studies of polymeric micelles indicated potent chemotherapeutic activity against premature orthotopic 4T1-luc breast cancer cells in vitro with prolonged survival [211]. The formulation reduced cancer stem-like cells fraction in these tumors, thereby extending the survival of mice. These findings indicate the potential of micelles in eradicating breast cancer cells and cancer stem-like cells, which will result in the prevention and recurrence of the cancer thereby improving patient survival.

Min et al. formulated and assessed MPEG-poly(β-amino ester)-based micelles encapsulated with camptothecin for breast tumor therapy. The pH of the tumor environment was the targeted site for the delivery of the loaded drug. The drug release was quick from the polymeric micelles in the acidic conditions simulating a tumor environment. The cytotoxicity results in vitro demonstrated that the plain polymeric micelles demonstrated significantly 100% cell viability of MDA-MB231 breast cancer cells even in higher concentrations confirming non-toxicity of micelles in the normal body. On the other hand, drug-loaded micelles showed low cell viability on MDA-MB231 breast cancer cells [212].

The extracellular pH at the solid tumor site is reported to be 6.8 when compared to the normal tissue with a pH of 7.4. Designing a pH-sensitive drug delivery is a good approach that can result in pH-responsiveness at the target tumor tissues. Most prepared pH-responsive delivery systems promote rapid drug release into the inner part of tumor cells [213,214]. However, endosomal pH-responsive micelles are not specific to the target cancer cells because the pH is the same as that of the normal cells endosomal acid which is below 6.5. There is still a need to improve the pH-responsiveness of micelles in weak acidic tumor microenvironment, and tumor accumulation in vivo.

Chida et al. formulated pH-responsive PEG-*b*-poly(β-benzyl l-aspartate) (PEG-*b*-PBLA)-based micelles encapsulated with anthracycline drug, epirubicin for breast cancer metastasis treatment. The in vitro drug release studies demonstrated that epirubicin was released from the polymeric micelles in a pH-dependent pattern with a low drug release mechanism at pH 7.4 (physiological condition). The epirubicin release kinetics was gradually fast with decreasing pH values until pH 6.7 (tumor microenvironment condition), and further increased at pH 5.5 (endosomal environment). The antitumor studies of polymeric micelles loaded with epirubicin against axillary lymph node metastasis (ALNM) of MDA-MB-231 TNBC demonstrated their potential to inhibit the growth of the spreading primary tumor and the growth of ALNM, through efficient drug activation promoted by the intratumoral acidic environment [215]. The release mechanism of the micelles inhibited the spread of cancer.

Gener and co-workers designed and evaluated micelles that are based on amphiphilic polymer Pluronic^®^ F127 loaded with Zileuton™ for breast cancer treatment. The in vivo and in vitro antitumor studies showed the drug-loaded polymeric micelles significant tumor growth inhibition in MDA-MB-231 and MCF-7 and breast cancer cell models when compared to the free Zileuton™. Furthermore, the polymeric micelles inhibited the circulation of breast tumor cells in the blood plasma effectively, and by doing so, significantly blocked metastatic spread [216]. The formulation eliminated cancer stem cells in the blood stream and reduced the number of cancer stem cells in the tumor in vivo. Eliminated cancer stem cells are usually replaced by new cells for tumor survival and propagation [217]. The capability of the micelles to eliminate cancer stem cell stream and metastatic spread is promising. These findings suggest that the formulation will show synergistic efficacy if combined with potent cytotoxic agents and is potentially a good approach to overcome cancer reoccurrence.

Lamch and co-workers formulated and evaluated in vitro the copolymers Pluronic-based micelles loaded with photofrin II^®^ for breast and ovarian cancer therapy. The research involved the evaluation of suitable conditions for photodynamic reaction and apoptosis in human cancer cells. The effect of Photofrin II^®^-loaded micelle formulation on the integrity of the cancer and human erythrocytes and cancer cells was also studied to determine the bioavailability of these nanocarriers in intravenous administration. The in vitro cytotoxicity studies of polymeric micelles exhibited superior pro-apoptotic and cytotoxic efficacy against MCF-7 tumor cells when compared to photofrin II^®^ [218].

Polymeric micelles are useful for efficient delivery of the loaded drug to the tumor cells and is also useful in reducing the effective drug concentration, which is suitable to diminish the side effects of the loaded anticancer drugs. The size of the micelles play a huge role in their uptake and formulation containing Photofrin II^®^ with the size below 20 nm were delivered inside the breast MCF-7/WT (caspase-3 deficient) cells efficiently. The results indicate that the administration of the formulation is potentially useful for the treatment of resistant cancers after irradiation.

Marcos and co-workers formulated and evaluated polymeric micelles that are based on poly(ethylene oxide)-poly(propylene oxide)-poly(ethylene oxide) triblock copolymers encapsulated with N-(2-hydroxyphenyl)-2-propylpentanamide for breast cancer targeting. The in vitro drug release kinetics of drug-entrapped micelles followed Weibull’s drug release model, demonstrating sustained drug release mechanism. The cytotoxicity effect of the polymeric micelles was significant in MDA-MB-231 [219]. N-(2-hydroxyphenyl)-2-propylpentanamide, has good anticancer activity against MDA-MB-231 cells but its low water solubility limit its therapeutic effectiveness. The incorporation of the drug into the micelles enhanced the water solubility by 23- and 31-fold. The hydrodynamic diameter of the formulation was 30 nm. The micelles improved the solubility of the drug forming stable aggregates and enhanced the drug release with a good maintenance. The sustained release system induced cell death and increased the drug administration time. Lu and co-workers prepared and evaluated micelles that are based on polylactide-*b*-(poly(2-hydroxyethyl acrylate-*co*-2-chloroethylmethacrylate-*co*-fluorescein-O-methacrylate)) (PLA-P(HEA-CEMA-F)) loaded with ruthenium complexes. The in vitro antitumor studies of the drug-loaded polymeric micelles in MDA-MB-231 and MCF-7 cancer cell models displayed enhanced anti-metastatic effect when compared to the free drugs and the tumor growth inhibition of drug-loaded micelles was 10 times higher when compared to the free drug [220]. Ruthenium complexes such as [Ru(η^6^-p-cymene)Cl_2_(PTA)] are not active against primary tumors but are effective in reducing metastasis. Their clearance rate from vital organs, their capability to reduce the progression of cancer in vivo, and their low toxicity make them useful for further studies [221]. The selectively targeted behavior of the micelle formulations on metastatic tumor cells limited the metastases of cancer.

Brinkman et al. formulated 12 amino acid peptide targeting epidermal growth factor receptor (EGFR) modified unimolecular PAMAM-PLA micelle nanoparticles loaded with aminoflavone for TNBC treatment. The in vitro cellular uptake studies showed that the modified polymeric micelles accumulated to a greater amount in the cytoplasm of MDA-MB-468 TNBC cells than the non-modified micelles. The in vitro cytotoxicity analysis demonstrated a significant decrease in cell viability of MDA-MB-468 and BT474 breast cancer cells that are incubated with modified micelle nanoparticles when compared to the unmodified micelles and free aminoflavone [222]. The micelles excellent stability and preferentially drug uptake at endosomal pH levels when compared to the blood pH suggest that they are potential therapeutics for EGFR-overexpressing triple negative breast cancer. Yang and co-workers formulated dendron-based micelles for topical delivery of endoxifen for estrogen-positive breast cancer treatment. Endoxifen is effective for the treatment and the prevention of estrogen-positive breast cancer; but its oral formulation induced severe side effects. The in vitro drug release profile demonstrated sustained release of endoxifen from the dendron-based micelles for 6 days. Micelles significantly improved the permeation of endoxifen through the mouse skin (up to 20-fold) and human skin (up to 4-fold). These results revealed that dendron-based micelles are useful for the topical delivery of endoxifen, providing a potential alternative administration method for chemoprevention of breast cancer [223]. Zhong and co-workers formulated cRGD-decorated, redox-activatable micellar mertansine prodrug to deliver mertansine to αvβ3 integrin overexpressed triple negative breast cancer. The cytotoxicity analysis showed that these micelles effectively suppressed MDA-MB-231 breast tumor growth without causing obvious side effects, as revealed by the body weight loss and histological analysis [224]. The formulation was effective in targeting and delivering of mertansine to α_v_β_3_ integrin overexpressing triple negative breast cancer with a potent tumor growth inhibition. These findings reveal the high affinity of the formulation to MDA-MB-231 cells and high intracellular drug release effective for potent antitumor effect; high stability and good targeting ability of the formulation resulting in high accumulation in the cancer cells; high effective inhibition of the tumor growth and reduced systemic toxicity when compared to the free mertansine showing its promising use for the treatment of triple negative breast cancer.

### 5.7. Limitations of Micelles

Micelles offer two distinct features when compared to other drug delivery systems, such as their small size (hydrodynamic sizes less than 50 nm) [225]. Its small particle sizes are suitable for good cellular uptake, blood circulation, tumor tissue penetration, and efficient internalization in cancer cells [225,226]. Their small size has improved the performance of the encapsulated drug in vivo due to the enhanced permeability and retention (EPR) effect [225,227]. They are also feasible for large-scale prooduction. It has well-defined molecular structures and good assembly behaviors. Micelle formulations are simple to produce at a large scale [228]. Polymeric micelles are useful for the delivery of hydrophobic and hydrophilic drugs to the target site. Their good stability, slow dissociation extend their circulation times, and their specific accumulation in the tumor tissues [229]. Small hydrophobic drugs are solubilized in the inner hydrophobic core of micelles, and their outer hydrophilic shell protects the drug from scavengers by the mononuclear phagocytic system [230,231]. Despite the distinct properties of micelles reported by several researchers, they are also limited due to their capability to undergo dilution resulting in dissociation when administered intravenously into the blood environment. The factor mentioned above is attributed to a shift from equilibrium to a unimer state and binding of the proteins to the unimeric components [231]. The protein binding can result in a burst release effect of the encapsulated drugs into the bloodstream, thereby nullifying the unique properties of micelles such as high drug loading, targeting capability, and prolonged blood circulation [225,229]. It can result in drug biodistribution that is favourable and toxicity with therapeutic outcomes similar to the free drugs [232]. Taxotere^®^, a micellar formulation displayed reduced therapeutic outcomes after intravenous administration resulting from its quick removal from the blood circulation [225].

The structural stability of micelles is affected by the physiological environment. Injection of micelles into the bloodstream results in them undergoing changes, including the disruption of the micellar structure via hydrolysis of the linkers etc. The disruption of the micellar causes a premature release of the encapsulated drug and the uptake of the drug in healthy tissues/organs, thereby inducing severe side effects and reducing the therapeutic efficacy of the loaded drug [233]. One strategic approach to improve the stability of micelles by cross-linking the micelles to afford a rigid micellar structure and prolonged blood circulation. Guo et al. reported mPEG-PDLA micelles with hydrophilic surface composed of PEG chain, which enhanced the micelles capability of escaping from the reticuloendothelial system, resulting in a slower clearance. A preferential accumulation of the formulation was significant in the tumors due to the enhanced permeability and prolonged circulation time [122]. Lang et al. coated the surface of the micelles with PEG, resulting in targeted drug delivery to the tumor and enhanced drug uptake [123]. Hu et al. reported Core-crosslinked polymeric micelles that promoted a 100% tumour-free survival [125]. Crosslinking of the micelles have resulted in them having distinct features such as prolonged circulation [122,123], efficient uptake into the tumor tissues with good retention [122,123,132], sustained drug release [125], and increased drug bioavailability [128,131,141].

## 6. Conclusions

This review reports the therapeutic biological outcomes (in vivo and in vitro) of dendrimers and micelles, as nanocarriers loaded with anticancer drugs on breast cancer. The nanocarriers exhibited distinct properties that revealed their capability to overcome the shortcomings of chemotherapeutic agents such as drug toxicity, multi-drug resistance, low drug cellular uptake, poor drug solubility, and drug bioavailability. The drug release profiles from the micelles and dendrimers were generally sustained at physiological conditions resulting in their low toxicity to the healthy cells and fast drug release at tumor conditions resulting in high cytotoxicity and uptake in the breast cancer cells. Furthermore, the polymeric nanocarriers exhibited high cellular uptake, important growth inhibition, inhibited metastases, and improved pharmacokinetic parameters (such as prolonged drug blood circulation). Although these systems show promising chemotherapeutic outcomes in breast tumor in vitro and in vivo in the reported preclinical studies, there is a serious need for further studies in order for these systems to reach clinical trial phases.

## Figures and Tables

**Table 1:** Classification of anticancer drugs and their side effects.

Anticancer Drugs	Drug Class	Mode of Action	Side Effects
Doxorubicin Epirubicin Camptothecin	Antitumor antibiotics	Binds to DNA and prevent DNA synthesis. They cause changes in the chromatin structure via inhibition of topoisomerase II.	Vomiting/nausea, weight loss, alopecia, thrombocytopenia, neutropenia, anorexia, and impaired immunity.
Cyclophosphamide (Cytoxan)	Alkylating agent Anti-neoplastic drug	Result in cross-linkage in DNA strands. Inhibit DNA biosynthesis and cell division.	Severe vomiting/nausea, neurotoxicity, pulmonary fibrosis, immune suppression, and alopecia.
Paclitaxel (taxol) Docetaxel (Taxotere)	Plant alkaloids Taxane class	Hinders microtubule disassembly and increase microtubule assembly. Terminate cell division in metaphase.	Leutropenia, hypersensitivity, anaphylaxis, thrombocytopenia, myalgias, fatigue, neutropenia, arthralgias, stomatitis, and Peripheral neuropathy
Vincristine	Vinca alkaloids Mitotic inhibitor	Binds to mitotic tubules and prevent the formation of microtubule in the mitotic spindle, inhibits mitosis I metaphase.	Mucositis, leukopenis, weight change, neurotoxicity, constipation, fatigue, secondary neoplasm, and thrombocytopenia
5-Fluorouracil	Pyrimidine antimetabolite	Inhibits enzyme formation needed for the synthesis of DNA.	Thrombocytopenia, peripheral neuropathy, mucositis, anaemia, neutropenia, neurotoxicity, cerebellar ataxia, and skin changes
Cisplatin Carboplatin	Platinum drugs	Inhibits the synthesis of DNA and prevents cell replication.	Ototoxicity, neurotoxicity, cardiotoxicity, nephrotoxicity, myelosuppression, neutropenia, and delayed hypersensitivity
Arimidex	aromatase inhibitor	Hinders aromatase, which intermediates transformation of androstenedione to estrone, which is then converted to estradiol.	Pain, mild nausea, vaginal dryness, osteoporosis, chest pain, increased risk for fractures, oedema, weakness, mild diarrhea, headache, and arthralgias
Tamoxifen	Selective estrogen receptor modulator	Strives with estrogen for receptor binding	Vaginal discharge, altered menses, amenorrhea, cough oedema, bone pain, musculoskeletal pain, dizziness, and endometrial hyperplasia.
Bevacizumab	Monoclonal antibody biological modifier	Binds to HER2 positive cancer cells and bring them up to be destroyed by the immune system. Cell proliferation inhibition.	Weakness, pain, flu-like symptoms, chills, diarrhea, abdominal pain, back pain, anorexia, congestive heart failure, and left ventricular cardiac dysfunction

**Table 2:** Clinical status of polymeric micelles and dendrimers nanoformulations.

Nanocarriers	Product/Trade Name	Copolymer Composition	Entrapped Drug	Cancer Therapy	Clinical Trial Phase	Ref
Micelles	Genexol^®^-PM/Cynviloq™	mPEG-PDLLA	Paclitaxel	Lung and Breast Cancer	Phase IV	[48,49]
Micelles	NK105	PEG-poly(aspartic acid) copolymer	Paclitaxel	Breast, colon, and gastric cancer	Phase III	[50]
Micelles	NK012	poly(l-glutamic acid)	Irinotecan	TNBC and small lung cancer	Phase II	[51]
Micelles	NC-6300	PEG-*b*-poly(aspartate-hydrazone)	Epirubicin	Breast and liver cancer	Phase I	[53]
Micelles	NK911	PEG-P (Asp)-DOX	Doxorubicin	Solid tumors	Phase II	[53]
Micelles	NC-4016	PEG-*b*-poly(β-glutamic acid)	Oxaliplatin	Solid Tumor	Phase I	[54]
Micelles	NC-6004	PEG-*b*-poly(l-glutamic acid)	Cisplatin	Pancreatic cancer	Phase III	[55]
Micelles	siRNA micelles	siRNA	siRNA	Lung Cancer	Phase I	[55]
Micelles	SP1049C	Pluronic L61 and F127	Doxorubicin	Adenocarcinoma	Phase III	[56]
Dendrimers	DEP^®^ docetaxel	PEGylated PLL	Docetaxel	breast, Lung, Prostate, and ovarian cancer	Phase I	[58]
Dendrimers	DEP^®^cabazitaxel	Polylysine	Cabazitaxel	Testicular, ovarian, breast, bladder, and head and neck	Phase I/11	[59]
Dendrimers	ImDendrim	Polylysine	188Rerhenium complex	Liver cancer	Phase I	[59]
Dendrimers	MAG-Tn3	Carbohydrate peptide lysine	Vaccine composed of tri Tn glycotope	Breast cancer	Phase I	[59]

**Table 3:** A summary of polymeric dendrimers nanocarriers loaded with anticancer drugs for breast targeting.

Polymers	Anticancer Drugs	Breast Cancer Models	Therapeutic Outcomes	References
PAMAM	Doxorubicin and cisplatin	MDA-MB-231 and MCF-7	The HA modified polymeric dendrimers showed good anticancer efficacy of when compared to the unmodified dendrimers	[73]
PAMAM	Doxorubicin	T47D and BT-549-Luc	High cellular uptake and binding.	[75]
pluronic F68- PAMAM	Doxorubicin	MCF-7/ADR	Improved antitumor activity	[76]
Collagen	Doxorubicin	MCF-7 and MDA-MB-231	Potential anticancer efficacy	[77]
PAMAM	Antisense oligodeoxynucleotides	MDA-MB-231	High cellular accumulation of the loaded drug.	[81]
polypropyleneimine	Oligodeoxynucleotide nanoparticles	MDA-MB-231	High cellular uptake	[82]
PAMAM	CpG oligodeoxynucleotide	SKBR3 and MDA-MB231	Decreased cell viability.	[83]
PAMAM	Antisense oligodeoxynucleotide	-	Growth tumor inhibition	[84]
PAMAM	Trastuzumab	MDA-MB-231and MDA-MB-453	Sustained drug release profile and reduced breast cancer cell viability.	[85]
polylysine	Trastuzumab	SKBR3 and MCF-7	High cellular internalization	[86]
PAMAM	Trastuzumab	MDA-MB-231 and SK-Br-3	Increased anticancer efficacy	[89]
PAMAM	Florescein isothiocyanated	4T1	Good cellular uptake	[90]
oligoethylene glycol	Tetrabromohydroquinone	MCF-7	Potent cytotoxicity efficacy against breast tumor.	[93]
PEG	Au nanoparticles	MCF-7	Excellent antitumor efficacy.	[94]
PAMAM	siRNA	SUM1315	High cellular uptake.	[95]
PAMAM	-	MDA-MB-231 and MCF-7	Good cytotoxicity	[97]
-	Curcumin	BT549 and SKBr3	Good anticancer activity	[98]
polylysine	PHSCN peptide	MDA-MB-231 and SUM-149	High cytotoxicity	[99]
PAMAM	Proscillaridin A and digoxin	MDA-MB-231 and MCF-7	High cell apoptosis	[101]
PAMAM	5-fluorouracil	MCF-7	Improved anticancer efficacy	[102]
PAMAM-NH2	-	MCF-7/ADR	Concentration-dependent cytotoxicity	[103]
PEG	Gemcitabine	4T1	Suppressed tumor volume	[104]
PAMAM	Tamoxifen	MCF-7	High cancer cell inhibitory effect	[105]

**Table 4:** A summary of micelles loaded with anticancer drugs.

Polymers	Drugs	Cancer Cell Lines	Therapeutic Outcomes	References
mPEG-PDLA	Docetaxel and resveratrol	MCF-7	Improved pharmacokinetic parameters, good biocompatibility, and safety.	[122]
BD-Peptide-PEG and BD-PEI	Docetaxel	4T1	High cellular uptake	[123]
Poly(d,l-lactide-*co*-2-methyl-2-carboxy-trimethylene carbonate)	Docetaxel	MDA-MB-231/H2N	High tumor growth inhibition and improvement in pharmacokinetic parameters	[124]
Monomethoxy PEG	Docetaxel	MDA-MB-231	Sustained in vitro drug release profile and potent in vivo antitumor efficacy.	[125]
PCL-*g*-PEI	Docetaxel and NIR dye	4T1	High tumor growth inhibition	[126]
TPGS	Docetaxel	MDA-MB-231 and MDA-MB-468	Greatly enhanced anticancer activity	[127]
PEG–PCL	Docetaxel	4T1	High tumor growth inhibition and decreased systemic toxicity	[128]
mPEG-polyester	Docetaxel	-	High tumor growth inhibition	[129]
mPEG_2000_-*b*-PDLLA_1300_	Docetaxel	4T1	greater anticancer activity	[130]
PLGA	Docetaxel	MDA-MB-231 and MCF-7	Improved antitumor activity	[131]
TPGS	Docetaxel	MDA-MB-231	Hindering of EGFR-overexpressing tumor cell lines	[132]
PLys-PPhe	Docetaxel	-	Improved tumor specificity	[133]
TPGS	Docetaxel	MDA-MB-231	High antitumor efficacy	[134]
MPEG-PDLLA-PLL	Docetaxel	4T1 and MCF-7	High tumor growth inhibition	[135]
PEG-*b*-PLGA	Docetaxel and chloroquine	MCF-7	Good anticancer activity	[136]
[NP(PEG750)(GlyPheLeu)_2_Et]_3_	Docetaxel	MDA-MB-231	Excellent anticancer efficacy	[137]
Poly(styrene maleic acid)-poly (amide-ether-ester-imide)	Docetaxel	MC4-L2	High tumor inhibition and increased survival in vivo	[138]
Polyvinyl caprolactam–polyvinyl acetate–PEG	Docetaxel and Fe_3_O_4_	MDA-MB-231 and MCF-7	Good anticancer activity	[139]
PEG-PLL-PLLeu	Docetaxel and siRNA-Bcl-2	MCF-7	Improved antitumor efficacy	[140]
mPEG2000- DSPE	Docetaxel	MCF-7	Better antitumor activity	[141]
HA	Doxorubicin	MCF-7	High tumor growth inhibition	[146]
Phis-PEG and PLLA-PEG	Doxorubicin	4T1	Moderate anticancer activity	[147]
Pluronic block copolymers	Doxorubicin	MDA-MB-231 and MDA-MB-468	Potent tumor growth inhibition	[148]
PEG-poly(aspartate hydrazide) block copolymers	Doxorubicin and wortmannin	MCF-7	Small particle size which is suitable for tumor-specific drug delivery	[149]
Carboxymethyl chitosan	Doxorubicin and cisplatin	HeLa	Synergistic anticancer effect	[150]
Dextran-retinoic acid	Doxorubicin	MDA-MB-468 and MCF-7	Good antitumor activity	[151]
PEG_2k_-PLA_5k_	Doxorubicin and curcumin	MCF-7/ADR	Higher tumor accumulation and tumor growth inhibitory effect	[152]
PEG-PCL-PEG	Doxorubicin	MCF-7	Suppressed tumor cells	[153]
-	Doxorubicin	MCF-7/ADR	Potential tumor growth inhibition	[154]
PLLA/PEG	Doxorubicin	MCF-7/DOX^R^	High cytotoxicity	[155]
Pluronic^®^ F127-poly (methyl-vinyl ether-alt-maleic acid) copolymer	Doxorubicin	MCF-7	Sustained drug release kinetics with good anticancer activity	[156]
PAA-*g*-PEG	Doxorubicin	4T1	High tumor accumulation	[157]
Poly (ε-caprolactone)-PEG	Doxorubicin	MCF-7	Time-delayed anticancer activity	[158]
PEG–PCL	Doxorubicin	MCF-7	High drug cellular uptake	[159]
Poly(ε-caprolactone)-polyphospho-ester	Doxorubicin	MCF-7	Improved antitumor activity	[160]
Dextran and indomethacin	Doxorubicin	MCF-7	Potential tumor growth inhibition	[161]
PEG–PCL	Doxorubicin and iron oxide	A431and MDA-MB-453	Significant tumor inhibitory effect	[162]
mPEG-PCL-*g*-cellulose	Doxorubicin	MCF7/ADR	Good cellular internalization	[163]
Poly 2,2-bis (methylol) propionic acid (bis-MPA)-PEG	Doxorubicin	MCF-7 and MDA-MB-468	Concentration-dependent anticancer activity	[164]
Poly (Îµ-caprolactone)	Doxorubicin	MCF-7	Higher antitumor activity	[165]
TPGS	Doxorubicin	MCF-7	100% long-term survival of the treated mice	[166]
mPEG-PLA	Doxorubicin and gemcitabine	MCF-7	Synergistic anticancer efficacy	[167]
Tetronic T1107, Pluronic F127, and TPGS	Doxorubicin	MDA-MB- 231	High anticancer efficacy	[168]
Pluronic	Doxorubicin and paclitaxel	MCF-7	Decreased cancer cell viability	[169]
MPEG-PCL-4-FBA	Doxorubicin and paclitaxel	MCF-7	Synergistic antitumor efficacy	[170]
Carboxymethyl chitosan	Paclitaxel	MCF-7	Improved oral drug bioavailability and synergistic anticancer activity.	[173]
Poly(2-oxazoline)-	Paclitaxel and cisplatin	LCC-6-MDR	Extended of the average lifespan of animals models in vivo	[174]
MPEG-*b*-P(LA-*co*-DHC/FA)	Paclitaxel	EMT-6	Reduced tumor mass and high tumor growth inhibition	[175]
Poly(2-ethyl-2-oxazoline)-poly(d,l-lactide)-	Paclitaxel and honokiol	MCF-7/ADR	Decreased cancer cell viability and small particle size which is beneficial for tumor-targeted drug delivery.	[176]
1,2-distearoyl-sn-glycero-3-phosphoethanol- amine-*N*-[methoxy(PEGl)-2000]	Paclitaxel	4T1	Great potential for theranostic application in the breast cancer therapy	[177]
Poly(ɛ-caprolactone)-PEG-poly(ɛ-caprolactone) triblock copolymers	Paclitaxel	EMT6	Low cell viability	[178]
PEG-*b*-PCL	Paclitaxel and salinomycin	MCF-7	High tumor growth inhibition	[179]
HA	paclitaxel and hydrophilic AURKA	MDA-MB- 231	Decreased tumor volume	[180]
PEG-PDLLA	Paclitaxel	MDA-MB-231	Significant tumor growth inhibition and cell apoptosis	[181]
Pluronic F127	Paclitaxel and lapatinib	T-47D	Suppressed proliferation of breast cancer cell	[182]
Soluplus^®^—Solutol^®^ HS15	Paclitaxel	MDA-MB-231	Good anticancer efficacy	[183]
HA	Paclitaxel	MCF-7	Good anticancer activity with reduced drug toxicity	[184]
PEG-PLA	Paclitaxel, 17-AAG, and Rapamycin	MDA-MB-231 and 549	High tumor growth inhibition	[185]
mPEG-*b*-poly(d,l-lactide)	Paclitaxel	-	Good clinical response rate	[186]
PEG-DSPE	Paclitaxel	MCF-7	Greater anticancer activity	[187]
PLGA-*g*-dextran	Paclitaxel	MCF-7	High anticancer efficacy	[188]
PPBV	Paclitaxel and curcumin	MCF-7	Small tumor volume	[189]
HA	Paclitaxel	4T1	Good cellular uptake	[190]
PEG-phosphatidyl-ethanolamine	Paclitaxel	MCF-7	High antitumor efficacy	[191]
Caprolactam–polyvinyl acetate–PEG and TPGS	Paclitaxel	MCF-7 and MDA-MB-231	Superior anticancer efficacy	[192]
Poly(β-amino ester)	Paclitaxel	MDA-MB-231	Good cellular uptake and suppression of the tumor metastasis	[193]
PEG-PE	Paclitaxel	MCF-7	Enhanced anticancer activity	[194]
mPEG-poly(capro-lactone	Paclitaxel and honokiol	4T1	High cellular uptake and increased anticancer efficacy	[195]
-	Paclitaxel	4T1	High growth inhibition on tumor metastasis	[196]
Pluronic	Paclitaxel	MCF-7	High anticancer activity	[197]
PEG	Paclitaxel	4T1.2	High anticancer activity	[198]
lipoprotein–N-succinyl chitosan–cystamine–urocanic acid	Paclitaxel and siRNA	MCF-7	Superior anticancer activity	[199]
phosphorylated calixarene	Curcumin	BT-549	Concentration-dependent cytotoxicity	[200]
PEG	Curcumin and doxorubicin	MCF-7/ADR	High synergistic anticancer activity	[201]
DSPE-PEG	Curcumin	MDA-MB-468	Decreased cancer cell viability	[202]
mPEG-b-PLA-	Curcumin and bortezomib	MDA-MB-231 and MCF-7	Maximum cellular uptake	[203]
MPEG-PCL	Curcumin	4T1	High anticancer activity	[204]
poly (2-oxazoline)	Cisplatin and paclitaxel	LCC-6-MDR	Prolonged plasma half-life and superior antitumor activity.	[205]
MPEG-block-poly (l-glutamic acid-*co*-l-phenylalanine)	Cisplatin	ZR-75-30	Inhibition of cancer cell proliferation	[206]
mPEG-*b*-poly(l-lactide-*co*-2-methyl-2-carboxyl-propylene carbonate	Platinum (II) drug	MCF-7	Dose-dependent cytotoxicity	[207]
MPEG-PCLA copolymer	Teniposide	MCF-7	Significant cell growth inhibition and reduced tumor volumes in vivo	[210]
PEG-*b*-poly(aspartate-hydrazide-epirubicin) copolymer	Epirubicin and staurosporine	orthotopic 4T1-luc	Potent anticancer efficacy and prolonged animal survival.	[211]
MPEG-poly(β-amino ester) copolymer	Camptothecin	MDA-MB231	Non-toxicity of the plain micelles and low cell viability for drug-loaded micelles	[212]
PEG-*b*-PBLA	Epirubicin	MDA-MB-231	Good tumor growth inhibition and suppression of ALNM	[215]
Pluronic^®^ F127	Zileuton™	MDA-MB-231 and MCF-7	Significant tumor growth inhibition and inhibition of metastatic spread and cancer cell blood circulation.	[216]
Pluronic	Photofrin II^®^	MCF-7	Improved in vitro pro-apoptotic and cytotoxic activity	[218]
Poly(ethylene oxide)-poly(propylene oxide)-poly(ethylene oxide) triblock copolymers	N-(2-Hydroxy-phenyl)-2-propyl-pentanamide	MDA-MB-231	Sustained drug release mechanism and anti-proliferative properties	[219]
PLA-P(HEA-CEMA-F)	Ruthenium complexes	MDA-MB-231 and MCF-7	Improved anti-metastatic effect and tumor growth inhibition	[220]
PAMAM-PLA	Aminoflavone	MDA-MB-468and BT474	Decreased cancer cell viability	[222]
Dendron	Endoxifen	-	Sustained drug release and improved permeation through skin	[223]
PEG	Mertansine	MDA-MB-231	Suppressed tumor growth	[224]
